# Supplementary material for: Menopausal hormone therapy and the female brain: Leveraging neuroimaging and prescription registry data from the UK Biobank cohort
Source: eLife. 2025 May 29;13:RP99538. doi: 10.7554/eLife.99538 (PMC12122002; doi:10.7554/eLife.99538)
Supplement: Supplementary file 1. [file elife-99538-supp1.docx]

**Supplemental File 1| Assessment of menopausal hormone therapy (MHT)-related variables in the UK Biobank (UKB).**

| **MHT variable** | **UKB Data-field** | **ACE touchscreen question** | **Coding** |
| --- | --- | --- | --- |
| Menopause | 2724 | “Have you had your menopause (periods stopped)?” | 1 (“Yes”) |
|  |  |  | 0 (“No”) |
|  |  |  | 2 («Not sure – had a hysterectomy») |
|  |  |  | 3 (“Not sure – other reason “) |
|  |  |  | -3 («Prefer not to answer») |
| Age at Menopause | 3581 | “How old were you when your periods stopped?” | Age in years |
|  |  |  | -1 («Do not know») |
|  |  |  | -3 («Prefer not to answer») |
| Bilateral Oophorectomy | 2834 | “Have you had BOTH ovaries removed?” | 1 (“Yes”) |
|  |  |  | 0 (“No”) |
|  |  |  | -5 («Not sure») |
|  |  |  | -3 («Prefer not to answer») |
| Hysterectomy | 3591 | “Have you had a hysterectomy (womb removed)?” | 1 (“Yes”) |
|  |  |  | 0 (“No”) |
|  |  |  | -5 («Not sure») |
|  |  |  | -3 («Prefer not to answer») |
| MHT use | 2814 | “Have you ever used hormone replacement therapy (HRT)?” | 1 (“Yes”) |
|  |  |  | 0 (“No”) |
|  |  |  | -1 («Do not know») |
|  |  |  | -3 («Prefer not to answer») |
| Age at last MHT use | 3546 | “How old were you when you last used HRT?” | Age in years |
|  |  |  | -1 («Do not know») |
|  |  |  | -11 (“Still taking HRT”) |
|  |  |  | -3 («Prefer not to answer») |
| Age at first MHT use | 3536 | “How old were you when you first used HRT?” | Age in years |
|  |  |  | -1 («Do not know») |
|  |  |  | -3 («Prefer not to answer») |
