# Supplementary material for: Menopausal hormone therapy and the female brain: Leveraging neuroimaging and prescription registry data from the UK Biobank cohort
Source: eLife. 2025 May 29;13:RP99538. doi: 10.7554/eLife.99538 (PMC12122002; doi:10.7554/eLife.99538)
Supplement: Supplementary file 2. [file elife-99538-supp2.docx]

**Supplemental File 2| Lifestyle factors, constituting the lifestyle score, in menopausal hormone therapy (MHT) never-, current, and past- users in the whole sample.**

|  | **MHT User Status** | | | **p-value** | | |
| --- | --- | --- | --- | --- | --- | --- |
|  | **Never** | **Current** | **Past** | **Never vs**  **Current** | **Never**  **vs Past** | **Current**  **vs Past** |
| **N** | 12,012 | 1,153 | 6,681 |  |  |  |
| **Age (years)** * | 61.6 ± 7.1 | 60.1 ± 6.8 | 67.5 ± 6.2 | **<0.001** | **<0.001** | **<0.001** |
| **Smoking Status, Yes, N(%)** | 313 (2.7) | 43 (3.8) | 187 (2.9) | **0.032** | 0.387 | 0.118 |
| **Alcohol Intake, N(%)** |  |  |  | **<0.001** | **<0.001** | **0.002** |
| Daily or almost daily | 1428 (12.1) | 181 (16.0) | 1012 (15.7) |  |  |  |
| Three or four times a week | 2957 (25.2) | 316 (28.0) | 1581 (24.5) |  |  |  |
| Once or twice a week | 3271 (27.8) | 306 (27.1) | 1662 (25.7) |  |  |  |
| One to three times a month | 1654 (14.1) | 141 (12.5) | 808 (12.5) |  |  |  |
| Special occasions only | 1572 (13.4) | 124 (11.0) | 905 (14.0) |  |  |  |
| Never | 873 (7.4) | 61 (5.4) | 495 (7.7) |  |  |  |
| **Time spend watching TV (hours)*** | 2.1 ± 3.0 | 2.1 ± 3.1 | 2.8 ± 2.5 | 0.720 | **<0.001** | **<0.001** |
| **Sleep duration (hours)*** | 7.1 ± 1.0 | 7.1 ± 1.1 | 7.1 ± 1.1 | 0.960 | 0.432 | 0.750 |
| **Fruit & Vegetable Intake (gram)*** | 685.1 ± 362.7 | 660.1 ± 342.1 | 706.0 ± 350.2 | **0.026** | **<0.001** | **<0.001** |
| **Oily Fish Intake, N(%)** |  |  |  | 0.202 | **<0.001** | **<0.001** |
| Never | 1156 (9.8) | 113 (10.0) | 444 (6.9) |  |  |  |
| Less than once a week | 3435 (29.2) | 321 (28.4) | 1568 (24.3) |  |  |  |
| Once a week | 4795 (40.8) | 431 (38.2) | 2823 (43.7) |  |  |  |
| 2-4 times a week | 2261 (19.2) | 252 (22.3) | 1572 (24.3) |  |  |  |
| 5-6 times a week | 82 (0.7) | 9 (0.8) | 43 (0.7) |  |  |  |
| Once or more daily | 26 (0.2) | 3 (0.3) | 13 (0.2) |  |  |  |
| **Beef Intake, N(%)** |  |  |  | 0.165 | **<0.001** | 0.053 |
| Never | 1851 (15.7) | 166 (14.7) | 800 (12.4) |  |  |  |
| Less than once a week | 5834 (49.6) | 529 (46.9) | 3323 (51.4) |  |  |  |
| Once a week | 3095 (26.3) | 327 (29.0) | 1750 (27.1) |  |  |  |
| 2-4 times a week | 966 (8.2) | 107 (9.5) | 582 (9.0) |  |  |  |
| 5-6 times a week | 8 (0.1) | 0 (0.0) | 6 (0.1) |  |  |  |
| Once or more daily | 1 (0.0) | 0 (0.0) | 2 (0.0) |  |  |  |
| **Pork Intake, N(%)** |  |  |  | 0.962 | **<0.001** | **0.041** |
| Never | 2708 (23.0) | 260 (23.0) | 1322 (20.5) |  |  |  |
| Less than once a week | 7114 (60.5) | 691 (61.2) | 3871 (59.9) |  |  |  |
| Once a week | 1703 (14.5) | 158 (14.0) | 1128 (17.5) |  |  |  |
| 2-4 times a week | 223 (1.9) | 20 (1.8) | 137 (2.1) |  |  |  |
| 5-6 times a week | 5 (0.0) | 0 (0.0) | 3 (0.0) |  |  |  |
| Once or more daily | 2 (0.0) | 0 (0.0) | 2 (0.0) |  |  |  |
| **Lamb/Mutton Intake, N(%)** |  |  |  | **0.048** | **<0.001** | 0.815 |
| Never | 3030 (25.8) | 261 (23.1) | 1418 (21.9) |  |  |  |
| Less than once a week | 7293 (62.0) | 699 (61.9) | 4043 (62.6) |  |  |  |
| Once a week | 1314 (11.2) | 155 (13.7) | 926 (14.3) |  |  |  |
| 2-4 times a week | 116 (1.0) | 14 (1.2) | 76 (1.2) |  |  |  |
| Once or more daily | 2 (0.0) | 0 (0.0) | 0 (0.0) |  |  |  |
| **Processed Meat Intake, N(%)** |  |  |  | 0.938 | **<0.001** | 0.073 |
| Never | 1741 (14.8) | 171 (15.1) | 803 (12.4) |  |  |  |
| Less than once a week | 4832 (41.1) | 465 (41.2) | 2815 (43.6) |  |  |  |
| Once a week | 2987 (25.4) | 280 (24.8) | 1661 (25.7) |  |  |  |
| 2-4 times a week | 2017 (17.2) | 198 (17.5) | 1086 (16.8) |  |  |  |
| 5-6 times a week | 147 (1.3) | 11 (1.0) | 87 (1.3) |  |  |  |
| Once or more daily | 31 (0.3) | 4 (0.4) | 11 (0.2) |  |  |  |
| **Moderate Activity (days/week)*** | 4.0 ± 2.3 | 3.9 ± 2.3 | 4.0 ± 2.4 | 0.126 | 0.051 | **0.018** |
| **Vigorous Activity (days/week)*** | 1.8 ± 1.8 | 1.8 ± 1.8 | 1.7 ± 1.9 | 0.312 | **<0.001** | 0.371 |

* Mean ± Standard Deviation. Fruit & Vegetable Intake is a composite of intake of dried fruits, fresh fruits, salad, raw vegetables and cooked vegetables. Smoking status take past and current smoking into account. Abbreviations: N, sample size. Significant differences between groups based on t/χ2 tests are highlighted in bold.
