# Supplementary material for: Menopausal hormone therapy and the female brain: Leveraging neuroimaging and prescription registry data from the UK Biobank cohort
Source: eLife. 2025 May 29;13:RP99538. doi: 10.7554/eLife.99538 (PMC12122002; doi:10.7554/eLife.99538)
Supplement: Supplementary file 3. [file elife-99538-supp3.docx]

**Supplemental File 3| Age prediction accuracy for the global grey and white matter models.**

| **Model** | **R^2^** | **RMSE** | **MAE** | ***r* [95% CI]** | ***p-value*** |
| --- | --- | --- | --- | --- | --- |
| GM | 0.53 ± 0.02 | 5.03 ± 0.09 | 4.02 ± 0.09 | 0.73 [0.73,0.74] | **< 0.001** |
| WM | 0.45 ± 0.03 | 5.40 ± 0.08 | 4.35 ± 0.08 | 0.67 [0.66, 0.68] | **< 0.001** |

Abbreviations: R^2^ **=** average R^2^, RMSE = root mean square error, MAE = mean absolute error, r = correlation between predicted and chronological age, CI = confidence interval.
