# Supplementary material for: Menopausal hormone therapy and the female brain: Leveraging neuroimaging and prescription registry data from the UK Biobank cohort
Source: eLife. 2025 May 29;13:RP99538. doi: 10.7554/eLife.99538 (PMC12122002; doi:10.7554/eLife.99538)
Supplement: Supplementary file 4. [file elife-99538-supp4.docx]

**Supplemental File 4| Associations between menopausal hormone therapy (MHT)-related variables and brain measures in the whole sample.**

| **MHT Variable** | **MRI Measure** | **beta** | **S.E.** | **t-value** | **p-value** | **pFDR-value** |
| --- | --- | --- | --- | --- | --- | --- |
| MHT Status | GM BAG | 0.034 | 0.008 | 3.986 | **6.74e-05** | **0.001** |
|  | WM BAG | 0.019 | 0.008 | 2.212 | **0.027** | 0.064 |
|  | Left Hippocampus | -0.021 | 0.008 | -2.726 | **0.006** | **0.020** |
|  | Right Hippocampus | -0.012 | 0.008 | -1.479 | 0.139 | 0.224 |
|  | WMH | 0.008 | 0.007 | 1.078 | 0.281 | 0.402 |
| Current MHT use | GM BAG | 0.218 | 0.038 | 5.782 | **7.54e-09** | **3.77e-07** |
|  | WM BAG | 0.155 | 0.038 | 4.101 | **4.13e-05** | **4.13e-04** |
|  | Left Hippocampus | -0.153 | 0.035 | -4.344 | **1.41e-05** | **2.11e-04** |
|  | Right Hippocampus | -0.130 | 0.035 | -3.674 | **2.39e-04** | **0.001** |
|  | WMH | -0.005 | 0.032 | -0.142 | 0.887 | 0.985 |
| Past MHT use | GM BAG | 0.040 | 0.021 | 1.916 | 0.055 | 0.111 |
|  | WM BAG | 0.012 | 0.021 | 0.574 | 0.566 | 0.707 |
|  | Left Hippocampus | -0.013 | 0.019 | -0.690 | 0.490 | 0.645 |
|  | Right Hippocampus | 0.000 | 0.019 | -0.012 | 0.990 | 0.992 |
|  | WMH | 0.022 | 0.018 | 1.263 | 0.207 | 0.313 |
| Age at first MHT use | GM BAG | 0.003 | 0.016 | 0.221 | 0.825 | 0.960 |
|  | WM BAG | 0.000 | 0.015 | -0.010 | 0.992 | 0.992 |
|  | Left Hippocampus | 0.010 | 0.015 | 0.669 | 0.503 | 0.645 |
|  | Right Hippocampus | -0.001 | 0.015 | -0.067 | 0.946 | 0.986 |
|  | WMH | -0.024 | 0.013 | -1.797 | 0.072 | 0.129 |
| Age at first MHT use relative  to age at menopause | GM BAG | 0.028 | 0.017 | 1.682 | 0.093 | 0.160 |
|  | WM BAG | 0.031 | 0.016 | 1.889 | 0.059 | 0.114 |
|  | Left Hippocampus | 0.002 | 0.016 | 0.098 | 0.922 | 0.986 |
|  | Right Hippocampus | -0.029 | 0.016 | -1.845 | 0.065 | 0.121 |
|  | WMH | -0.007 | 0.014 | -0.494 | 0.622 | 0.758 |
| Age at last MHT use | GM BAG | 0.046 | 0.018 | 2.550 | **0.011** | **0.030** |
|  | WM BAG | 0.039 | 0.018 | 2.165 | **0.030** | 0.069 |
|  | Left Hippocampus | -0.036 | 0.018 | -2.036 | **0.042** | 0.091 |
|  | Right Hippocampus | -0.027 | 0.017 | -1.565 | 0.118 | 0.196 |
|  | WMH | 0.019 | 0.016 | 1.211 | 0.226 | 0.332 |
| Age at last MHT use relative  to age at menopause | GM BAG | 0.062 | 0.018 | 3.413 | **0.001** | **0.003** |
|  | WM BAG | 0.064 | 0.018 | 3.505 | **4.63e-04** | **0.002** |
|  | Left Hippocampus | -0.055 | 0.018 | -3.100 | **0.002** | **0.008** |
|  | Right Hippocampus | -0.056 | 0.018 | -3.205 | **0.001** | **0.006** |
|  | WMH | 0.039 | 0.016 | 2.440 | **0.015** | **0.039** |
| Duration of MHT use | GM BAG | 0.072 | 0.016 | 4.454 | **8.67e-06** | **2.11e-04** |
|  | WM BAG | 0.062 | 0.016 | 3.853 | **1.19e-04** | **0.001** |
|  | Left Hippocampus | -0.065 | 0.015 | -4.307 | **1.69e-05** | **2.11e-04** |
|  | Right Hippocampus | -0.044 | 0.015 | -2.915 | **0.004** | **0.012** |
|  | WMH | 0.027 | 0.014 | 1.924 | 0.054 | 0.111 |
| Bilateral Oophorectomy | GM BAG | -0.033 | 0.013 | -2.594 | **0.009** | **0.028** |
|  | WM BAG | -0.004 | 0.013 | -0.291 | 0.771 | 0.918 |
|  | Left Hippocampus | 0.012 | 0.012 | 1.046 | 0.296 | 0.411 |
|  | Right Hippocampus | -0.001 | 0.012 | -0.076 | 0.939 | 0.986 |
|  | WMH | 0.002 | 0.011 | 0.164 | 0.869 | 0.985 |
| Hysterectomy | GM BAG | -0.047 | 0.013 | -3.497 | **4.74e-04** | **0.002** |
|  | WM BAG | -0.019 | 0.014 | -1.401 | 0.161 | 0.252 |
|  | Left Hippocampus | 0.038 | 0.013 | 2.990 | **0.003** | **0.010** |
|  | Right Hippocampus | 0.030 | 0.013 | 2.406 | **0.016** | **0.040** |
|  | WMH | -0.012 | 0.012 | -0.991 | 0.322 | 0.435 |

Significant results are highlighted in bold. False discovery rate (FDR) correction was applied across all brain measures and MHT variables listed in this table. Abbreviations: MRI = magnetic resonance imaging, S.E. = standard error, GM = grey matter, BAG = brain age gap, WM = white matter, WMH = white matter hyperintensity.
