# Supplementary material for: Menopausal hormone therapy and the female brain: Leveraging neuroimaging and prescription registry data from the UK Biobank cohort
Source: eLife. 2025 May 29;13:RP99538. doi: 10.7554/eLife.99538 (PMC12122002; doi:10.7554/eLife.99538)
Supplement: Supplementary file 5. [file elife-99538-supp5.docx]

**Supplemental File 5| Associations between menopausal hormone therapy (MHT)-related variables and brain measures in the prescription MHT sample.**

| **MHT Variable** | **MRI Measure** | **beta** | **S.E.** | **t-value** | **p-value** | **pFDR-value** |
| --- | --- | --- | --- | --- | --- | --- |
| **MHT formulation** |  |  |  |  |  |  |
| Estrogens-only | GM BAG | -0.025 | 0.068 | -0.367 | 0.714 | 0.994 |
|  | WM BAG | 0.040 | 0.068 | 0.591 | 0.554 | 0.994 |
|  | Left Hippocampus | -0.066 | 0.064 | -1.032 | 0.302 | 0.994 |
|  | Right Hippocampus | -0.050 | 0.064 | -0.775 | 0.438 | 0.994 |
|  | WMH | 0.017 | 0.060 | 0.283 | 0.777 | 0.994 |
| Estrogens+Progestin | GM BAG | 0.005 | 0.057 | 0.093 | 0.926 | 0.994 |
|  | WM BAG | 0.127 | 0.057 | 2.221 | **0.026** | 0.832 |
|  | Left Hippocampus | -0.010 | 0.054 | -0.179 | 0.858 | 0.994 |
|  | Right Hippocampus | -0.011 | 0.054 | -0.198 | 0.843 | 0.994 |
|  | WMH | 0.052 | 0.050 | 1.039 | 0.299 | 0.994 |
| **Route of Administration** |  |  |  |  |  |  |
| oral | GM BAG | -0.048 | 0.066 | -0.729 | 0.466 | 0.994 |
|  | WM BAG | 0.113 | 0.066 | 1.708 | 0.088 | 0.994 |
|  | Left Hippocampus | -0.030 | 0.063 | -0.478 | 0.632 | 0.994 |
|  | Right Hippocampus | -0.001 | 0.063 | -0.023 | 0.981 | 0.994 |
|  | WMH | 0.134 | 0.057 | 2.336 | **0.019** | 0.832 |
| transdermal | GM BAG | -0.205 | 0.125 | -1.636 | 0.102 | 0.994 |
|  | WM BAG | -0.038 | 0.126 | -0.300 | 0.764 | 0.994 |
|  | Left Hippocampus | -0.040 | 0.118 | -0.338 | 0.735 | 0.994 |
|  | Right Hippocampus | -0.053 | 0.118 | -0.448 | 0.654 | 0.994 |
|  | WMH | -0.094 | 0.110 | -0.852 | 0.394 | 0.994 |
| vaginal | GM BAG | 0.138 | 0.096 | 1.432 | 0.152 | 0.994 |
|  | WM BAG | 0.143 | 0.096 | 1.485 | 0.138 | 0.994 |
|  | Left Hippocampus | -0.052 | 0.091 | -0.571 | 0.568 | 0.994 |
|  | Right Hippocampus | -0.002 | 0.091 | -0.022 | 0.983 | 0.994 |
|  | WMH | 0.070 | 0.086 | 0.818 | 0.414 | 0.994 |
| injection | GM BAG | 0.446 | 0.333 | 1.338 | 0.181 | 0.994 |
|  | WM BAG | 0.204 | 0.333 | 0.612 | 0.541 | 0.994 |
|  | Left Hippocampus | -0.276 | 0.315 | -0.876 | 0.381 | 0.994 |
|  | Right Hippocampus | 0.102 | 0.315 | 0.323 | 0.747 | 0.994 |
|  | WMH | -0.072 | 0.286 | -0.251 | 0.802 | 0.994 |
| mixed | GM BAG | 0.012 | 0.091 | 0.128 | 0.898 | 0.994 |
|  | WM BAG | 0.062 | 0.091 | 0.680 | 0.496 | 0.994 |
|  | Left Hippocampus | -0.001 | 0.086 | -0.007 | 0.994 | 0.994 |
|  | Right Hippocampus | -0.093 | 0.086 | -1.082 | 0.279 | 0.994 |
|  | WMH | -0.101 | 0.079 | -1.274 | 0.203 | 0.994 |
| **Estrogen-only Forms** |  |  |  |  |  |  |
| Bioidentical | GM BAG | -0.013 | 0.076 | -0.169 | 0.866 | 0.994 |
|  | WM BAG | 0.043 | 0.076 | 0.560 | 0.575 | 0.994 |
|  | Left Hippocampus | -0.041 | 0.072 | -0.563 | 0.573 | 0.994 |
|  | Right Hippocampus | -0.013 | 0.072 | -0.180 | 0.857 | 0.994 |
|  | WMH | 0.001 | 0.067 | 0.012 | 0.991 | 0.994 |
| Synthetic | GM BAG | 0.053 | 0.214 | 0.248 | 0.804 | 0.994 |
|  | WM BAG | 0.028 | 0.214 | 0.133 | 0.894 | 0.994 |
|  | Left Hippocampus | -0.125 | 0.202 | -0.622 | 0.534 | 0.994 |
|  | Right Hippocampus | -0.066 | 0.202 | -0.326 | 0.744 | 0.994 |
|  | WMH | 0.106 | 0.184 | 0.580 | 0.562 | 0.994 |
| **Estrogen-only,**  **active ingredient** |  |  |  |  |  |  |
| estradiol | GM BAG | -0.231 | 0.172 | -1.343 | 0.179 | 0.994 |
|  | WM BAG | -0.133 | 0.172 | -0.771 | 0.441 | 0.994 |
|  | Left Hippocampus | -0.049 | 0.162 | -0.303 | 0.762 | 0.994 |
|  | Right Hippocampus | 0.020 | 0.162 | 0.121 | 0.904 | 0.994 |
|  | WMH | -0.050 | 0.150 | -0.336 | 0.737 | 0.994 |
| estradiol hemihydrate | GM BAG | 0.039 | 0.085 | 0.465 | 0.642 | 0.994 |
|  | WM BAG | 0.085 | 0.085 | 1.004 | 0.315 | 0.994 |
|  | Left Hippocampus | -0.038 | 0.080 | -0.479 | 0.632 | 0.994 |
|  | Right Hippocampus | -0.021 | 0.080 | -0.258 | 0.796 | 0.994 |
|  | WMH | 0.013 | 0.075 | 0.178 | 0.859 | 0.994 |
| estradiol valerate | GM BAG | 0.478 | 0.500 | 0.957 | 0.338 | 0.994 |
|  | WM BAG | 0.259 | 0.500 | 0.517 | 0.605 | 0.994 |
|  | Left Hippocampus | -0.024 | 0.472 | -0.051 | 0.960 | 0.994 |
|  | Right Hippocampus | 0.388 | 0.472 | 0.823 | 0.410 | 0.994 |
|  | WMH | 0.188 | 0.429 | 0.438 | 0.662 | 0.994 |
| CEE | GM BAG | -0.042 | 0.236 | -0.179 | 0.858 | 0.994 |
|  | WM BAG | -0.022 | 0.236 | -0.095 | 0.925 | 0.994 |
|  | Left Hippocampus | -0.148 | 0.223 | -0.662 | 0.508 | 0.994 |
|  | Right Hippocampus | -0.167 | 0.223 | -0.747 | 0.455 | 0.994 |
|  | WMH | 0.088 | 0.203 | 0.433 | 0.665 | 0.994 |
| Mixed | GM BAG | -0.177 | 0.193 | -0.914 | 0.361 | 0.994 |
|  | WM BAG | 0.030 | 0.193 | 0.153 | 0.878 | 0.994 |
|  | Left Hippocampus | -0.181 | 0.182 | -0.995 | 0.320 | 0.994 |
|  | Right Hippocampus | -0.269 | 0.182 | -1.479 | 0.139 | 0.994 |
|  | WMH | 0.039 | 0.169 | 0.232 | 0.816 | 0.994 |
| **Estrogens-only,**  **Dosage (mg)** |  |  |  |  |  |  |
|  | GM BAG | -0.045 | 0.074 | -0.605 | 0.546 | 0.994 |
|  | WM BAG | -0.080 | 0.075 | -1.065 | 0.288 | 0.994 |
|  | Left Hippocampus | -0.004 | 0.071 | -0.051 | 0.960 | 0.994 |
|  | Right Hippocampus | -0.018 | 0.071 | -0.256 | 0.798 | 0.994 |
|  | WMH | -0.007 | 0.061 | -0.108 | 0.914 | 0.994 |
| **Estrogens-only,**  **Duration of Use (weeks)** |  |  |  |  |  |  |
|  | GM BAG | -0.047 | 0.081 | -0.578 | 0.564 | 0.994 |
|  | WM BAG | -0.141 | 0.085 | -1.648 | 0.101 | 0.994 |
|  | Left Hippocampus | -0.007 | 0.083 | -0.090 | 0.928 | 0.994 |
|  | Right Hippocampus | -0.037 | 0.079 | -0.475 | 0.635 | 0.994 |
|  | WMH | -0.146 | 0.066 | -2.221 | **0.028** | 0.832 |
| **Estrogens + Progestins Form** |  |  |  |  |  |  |
| Bioidentical | GM BAG | -0.119 | 0.249 | -0.477 | 0.633 | 0.994 |
|  | WM BAG | 0.011 | 0.250 | 0.044 | 0.965 | 0.994 |
|  | Left Hippocampus | 0.108 | 0.236 | 0.457 | 0.647 | 0.994 |
|  | Right Hippocampus | 0.336 | 0.236 | 1.424 | 0.154 | 0.994 |
|  | WMH | 0.034 | 0.215 | 0.157 | 0.875 | 0.994 |
| Synthetic | GM BAG | -0.025 | 0.186 | -0.133 | 0.895 | 0.994 |
|  | WM BAG | 0.204 | 0.186 | 1.093 | 0.275 | 0.994 |
|  | Left Hippocampus | 0.134 | 0.176 | 0.760 | 0.447 | 0.994 |
|  | Right Hippocampus | 0.162 | 0.176 | 0.921 | 0.357 | 0.994 |
|  | WMH | 0.135 | 0.160 | 0.844 | 0.399 | 0.994 |
| Bioidentical & Synthetic | GM BAG | 0.009 | 0.105 | 0.089 | 0.929 | 0.994 |
|  | WM BAG | 0.057 | 0.105 | 0.544 | 0.587 | 0.994 |
|  | Left Hippocampus | -0.097 | 0.099 | -0.979 | 0.327 | 0.994 |
|  | Right Hippocampus | -0.115 | 0.099 | -1.158 | 0.247 | 0.994 |
|  | WMH | 0.075 | 0.091 | 0.819 | 0.413 | 0.994 |
| **Estrogens + Progestins,**  **active ingredient** |  |  |  |  |  |  |
| estradiol hemihydrate &  norethisterone acetate | GM BAG | -0.067 | 0.140 | -0.481 | 0.631 | 0.994 |
|  | WM BAG | -0.022 | 0.140 | -0.155 | 0.877 | 0.994 |
|  | Left Hippocampus | 0.045 | 0.132 | 0.338 | 0.735 | 0.994 |
|  | Right Hippocampus | -0.005 | 0.132 | -0.041 | 0.967 | 0.994 |
|  | WMH | 0.062 | 0.122 | 0.509 | 0.610 | 0.994 |
| estradiol hemihydrate &  dydrogesterone | GM BAG | -0.200 | 0.267 | -0.749 | 0.454 | 0.994 |
|  | WM BAG | -0.093 | 0.267 | -0.348 | 0.728 | 0.994 |
|  | Left Hippocampus | 0.036 | 0.252 | 0.144 | 0.886 | 0.994 |
|  | Right Hippocampus | 0.321 | 0.252 | 1.271 | 0.204 | 0.994 |
|  | WMH | -0.009 | 0.229 | -0.040 | 0.968 | 0.994 |
| estradiol hemihydrate &  norethisterone | GM BAG | 0.277 | 0.277 | 1.001 | 0.317 | 0.994 |
|  | WM BAG | 0.115 | 0.277 | 0.414 | 0.679 | 0.994 |
|  | Left Hippocampus | -0.368 | 0.262 | -1.406 | 0.160 | 0.994 |
|  | Right Hippocampus | -0.443 | 0.262 | -1.693 | 0.091 | 0.994 |
|  | WMH | 0.354 | 0.238 | 1.488 | 0.137 | 0.994 |
| CEE & norgestrel | GM BAG | -0.076 | 0.229 | -0.331 | 0.741 | 0.994 |
|  | WM BAG | -0.026 | 0.230 | -0.113 | 0.910 | 0.994 |
|  | Left Hippocampus | 0.134 | 0.217 | 0.619 | 0.536 | 0.994 |
|  | Right Hippocampus | 0.036 | 0.217 | 0.164 | 0.869 | 0.994 |
|  | WMH | 0.136 | 0.197 | 0.689 | 0.491 | 0.994 |
| CEE &  medroxyprogesterone acetate | GM BAG | 0.074 | 0.316 | 0.234 | 0.815 | 0.994 |
|  | WM BAG | 0.634 | 0.316 | 2.004 | **0.045** | 0.994 |
|  | Left Hippocampus | 0.128 | 0.298 | 0.429 | 0.668 | 0.994 |
|  | Right Hippocampus | 0.399 | 0.298 | 1.336 | 0.182 | 0.994 |
|  | WMH | 0.130 | 0.271 | 0.481 | 0.631 | 0.994 |
| tibolone | GM BAG | -0.441 | 0.277 | -1.592 | 0.111 | 0.994 |
|  | WM BAG | 0.025 | 0.278 | 0.090 | 0.929 | 0.994 |
|  | Left Hippocampus | 0.033 | 0.262 | 0.126 | 0.900 | 0.994 |
|  | Right Hippocampus | 0.166 | 0.262 | 0.633 | 0.526 | 0.994 |
|  | WMH | -0.180 | 0.248 | -0.725 | 0.469 | 0.994 |
| Mixed | GM BAG | 0.067 | 0.079 | 0.854 | 0.393 | 0.994 |
|  | WM BAG | 0.179 | 0.079 | 2.270 | **0.023** | 0.832 |
|  | Left Hippocampus | -0.002 | 0.075 | -0.030 | 0.976 | 0.994 |
|  | Right Hippocampus | -0.035 | 0.075 | -0.476 | 0.634 | 0.994 |
|  | WMH | 0.037 | 0.069 | 0.536 | 0.592 | 0.994 |
| **Estrogens + Progestins,**  **Progestin Generation** |  |  |  |  |  |  |
| 1stGen | GM BAG | 0.054 | 0.094 | 0.579 | 0.563 | 0.994 |
|  | WM BAG | 0.139 | 0.094 | 1.485 | 0.138 | 0.994 |
|  | Left Hippocampus | -0.044 | 0.088 | -0.499 | 0.618 | 0.994 |
|  | Right Hippocampus | -0.039 | 0.088 | -0.439 | 0.661 | 0.994 |
|  | WMH | 0.088 | 0.082 | 1.079 | 0.281 | 0.994 |
| 2ndGen | GM BAG | -0.004 | 0.156 | -0.025 | 0.980 | 0.994 |
|  | WM BAG | 0.068 | 0.157 | 0.432 | 0.666 | 0.994 |
|  | Left Hippocampus | 0.083 | 0.148 | 0.564 | 0.573 | 0.994 |
|  | Right Hippocampus | 0.105 | 0.148 | 0.714 | 0.475 | 0.994 |
|  | WMH | 0.119 | 0.135 | 0.883 | 0.377 | 0.994 |
| **Estrogens + Progestins,**  **Dosage (mg)** |  |  |  |  |  |  |
| Estrogens | GM BAG | -0.068 | 0.058 | -1.185 | 0.237 | 0.994 |
|  | WM BAG | -0.004 | 0.058 | -0.077 | 0.938 | 0.994 |
|  | Left Hippocampus | -0.053 | 0.056 | -0.950 | 0.343 | 0.994 |
|  | Right Hippocampus | 0.001 | 0.056 | 0.016 | 0.987 | 0.994 |
|  | WMH | 0.063 | 0.053 | 1.192 | 0.234 | 0.994 |
| Progestins | GM BAG | 0.062 | 0.057 | 1.099 | 0.273 | 0.994 |
|  | WM BAG | 0.039 | 0.057 | 0.684 | 0.495 | 0.994 |
|  | Left Hippocampus | 0.072 | 0.055 | 1.292 | 0.197 | 0.994 |
|  | Right Hippocampus | 0.055 | 0.055 | 0.984 | 0.326 | 0.994 |
|  | WMH | 0.071 | 0.051 | 1.390 | 0.166 | 0.994 |
| **Estrogens + Progestins,**  **Duration of Use (weeks)** | |  |  |  |  |  |
| Estrogens | GM BAG | 0.089 | 0.071 | 1.247 | 0.213 | 0.994 |
|  | WM BAG | -0.022 | 0.073 | -0.297 | 0.767 | 0.994 |
|  | Left Hippocampus | -0.149 | 0.069 | -2.165 | **0.031** | 0.832 |
|  | Right Hippocampus | -0.165 | 0.068 | -2.438 | **0.015** | 0.832 |
|  | WMH | -0.093 | 0.066 | -1.417 | 0.158 | 0.994 |
| Progestins | GM BAG | -0.015 | 0.074 | -0.207 | 0.836 | 0.994 |
|  | WM BAG | 0.071 | 0.076 | 0.938 | 0.349 | 0.994 |
|  | Left Hippocampus | -0.026 | 0.072 | -0.368 | 0.713 | 0.994 |
|  | Right Hippocampus | 0.021 | 0.071 | 0.295 | 0.768 | 0.994 |
|  | WMH | 0.045 | 0.068 | 0.654 | 0.514 | 0.994 |

Significant results are highlighted in bold. False discovery rate (FDR) correction was applied across all brain measures and MHT variables listed in this table. Abbreviations: MRI = magnetic resonance imaging, S.E. = standard error, GM = grey matter, BAG = brain age gap, WM = white matter, WMH = white matter hyperintensity, CEE = conjugated equine estrogen, Gen = generation.
