# Supplementary material for: Menopausal hormone therapy and the female brain: Leveraging neuroimaging and prescription registry data from the UK Biobank cohort
Source: eLife. 2025 May 29;13:RP99538. doi: 10.7554/eLife.99538 (PMC12122002; doi:10.7554/eLife.99538)
Supplement: Supplementary file 6. [file elife-99538-supp6.docx]

**Supplemental File 6| Associations between APOE ε4 genotype and brain measures in the whole sample.**

| **Genotype** | **MRI Measure** | **beta** | **S.E.** | **t-value** | **p-value** | **pFDR-value** |
| --- | --- | --- | --- | --- | --- | --- |
| APOE ε4 | GM BAG | 0.017 | 0.008 | 2.060 | **0.039** | 0.118 |
| ε3/ε4 | GM BAG | 0.040 | 0.019 | 2.070 | **0.038** | 0.118 |
| ε4/ε4 | GM BAG | 0.022 | 0.053 | 0.422 | 0.673 | 0.721 |
| APOE ε4 | WM BAG | 0.011 | 0.008 | 1.372 | 0.170 | 0.252 |
| ε3/ε4 | WM BAG | 0.027 | 0.019 | 1.382 | 0.167 | 0.252 |
| ε4/ε4 | WM BAG | 0.014 | 0.053 | 0.270 | 0.787 | 0.787 |
| APOE ε4 | Left Hippocampus | -0.019 | 0.008 | -2.467 | **0.014** | 0.068 |
| ε3/ε4 | Left Hippocampus | -0.034 | 0.018 | -1.865 | 0.062 | 0.138 |
| ε4/ε4 | Left Hippocampus | -0.134 | 0.050 | -2.699 | **0.007** | 0.052 |
| APOE ε4 | Right Hippocampus | -0.010 | 0.008 | -1.327 | 0.185 | 0.252 |
| ε3/ε4 | Right Hippocampus | -0.010 | 0.018 | -0.531 | 0.595 | 0.687 |
| ε4/ε4 | Right Hippocampus | -0.156 | 0.050 | -3.135 | **0.002** | **0.026** |
| APOE ε4 | WMH | 0.013 | 0.007 | 1.849 | 0.064 | 0.138 |
| ε3/ε4 | WMH | 0.027 | 0.017 | 1.640 | 0.101 | 0.189 |
| ε4/ε4 | WMH | 0.053 | 0.046 | 1.154 | 0.249 | 0.311 |

Significant results are highlighted in bold. False discovery rate (FDR) correction was applied across all brain measures and MHT variables listed in this table. Abbreviations: APOE = apolipoprotein, MRI = magnetic resonance imaging, S.E. = standard error, GM = grey matter, BAG = brain age gap, WM = white matter, WMH = white matter hyperintensity.
