# Supplementary material for: Menopausal hormone therapy and the female brain: Leveraging neuroimaging and prescription registry data from the UK Biobank cohort
Source: eLife. 2025 May 29;13:RP99538. doi: 10.7554/eLife.99538 (PMC12122002; doi:10.7554/eLife.99538)
Supplement: Supplementary file 7. [file elife-99538-supp7.docx]

**Supplemental File 7| Interactions between APOE ε4 genotype and menopausal hormone therapy (MHT)-related variables on brain measures in the entire sample.**

| **MHT Variable** | **MRI Measure** | **beta** | **S.E.** | **t-value** | **p-value** | **pFDR-value** |
| --- | --- | --- | --- | --- | --- | --- |
| **MHT Status * APOE ε4** | GM BAG | 0.009 | 0.008 | 1.050 | 0.294 | 0.927 |
|  | WM BAG | 0.007 | 0.008 | 0.796 | 0.426 | 0.927 |
|  | Left Hippocampus | -0.008 | 0.008 | -1.089 | 0.276 | 0.927 |
|  | Right Hippocampus | -0.010 | 0.008 | -1.344 | 0.179 | 0.927 |
|  | WMH | -0.001 | 0.007 | -0.150 | 0.880 | 0.945 |
| **Current MHT use * APOE ε4** | GM BAG | 0.047 | 0.040 | 1.189 | 0.234 | 0.927 |
|  | WM BAG | 0.014 | 0.040 | 0.363 | 0.717 | 0.927 |
|  | Left Hippocampus | 0.029 | 0.037 | 0.772 | 0.440 | 0.927 |
|  | Right Hippocampus | 0.047 | 0.037 | 1.275 | 0.202 | 0.927 |
|  | WMH | -0.006 | 0.034 | -0.170 | 0.865 | 0.945 |
| **Past MHT use * APOE ε4** | GM BAG | 0.013 | 0.020 | 0.637 | 0.524 | 0.927 |
|  | WM BAG | 0.006 | 0.020 | 0.281 | 0.778 | 0.927 |
|  | Left Hippocampus | -0.031 | 0.019 | -1.639 | 0.101 | 0.927 |
|  | Right Hippocampus | -0.037 | 0.019 | -1.963 | 0.050 | 0.927 |
|  | WMH | -0.001 | 0.018 | -0.083 | 0.934 | 0.945 |
| **Age at first MHT use * APOE ε4** | GM BAG | 0.018 | 0.016 | 1.088 | 0.277 | 0.927 |
|  | WM BAG | 0.006 | 0.016 | 0.377 | 0.706 | 0.927 |
|  | Left Hippocampus | 0.013 | 0.015 | 0.813 | 0.416 | 0.927 |
|  | Right Hippocampus | 0.008 | 0.015 | 0.534 | 0.593 | 0.927 |
|  | WMH | 0.006 | 0.014 | 0.441 | 0.660 | 0.927 |
| **Age at first MHT use relative**  **to age at menopause**  *** APOE ε4** | GM BAG | -0.017 | 0.019 | -0.899 | 0.369 | 0.927 |
|  | WM BAG | 0.006 | 0.019 | 0.331 | 0.741 | 0.927 |
|  | Left Hippocampus | -0.004 | 0.018 | -0.207 | 0.836 | 0.945 |
|  | Right Hippocampus | -0.006 | 0.018 | -0.344 | 0.731 | 0.927 |
|  | WMH | -0.026 | 0.016 | -1.604 | 0.109 | 0.927 |
| **Age at last MHT use**  *** APOE** | GM BAG | 0.015 | 0.018 | 0.814 | 0.416 | 0.927 |
|  | WM BAG | -0.007 | 0.018 | -0.397 | 0.691 | 0.927 |
|  | Left Hippocampus | 0.010 | 0.018 | 0.558 | 0.577 | 0.927 |
|  | Right Hippocampus | -0.002 | 0.017 | -0.092 | 0.927 | 0.945 |
|  | WMH | -0.022 | 0.016 | -1.383 | 0.167 | 0.927 |
| **Age at last MHT use relative**  **to age at menopause**  *** APOE ε4** | GM BAG | -0.007 | 0.017 | -0.437 | 0.662 | 0.927 |
|  | WM BAG | 0.015 | 0.017 | 0.868 | 0.386 | 0.927 |
|  | Left Hippocampus | -0.003 | 0.016 | -0.202 | 0.840 | 0.945 |
|  | Right Hippocampus | -0.006 | 0.016 | -0.386 | 0.699 | 0.927 |
|  | WMH | -0.008 | 0.014 | -0.561 | 0.575 | 0.927 |
| **Duration of MHT use**  *** APOE ε4** | GM BAG | 0.006 | 0.015 | 0.359 | 0.720 | 0.927 |
|  | WM BAG | -0.008 | 0.015 | -0.534 | 0.594 | 0.927 |
|  | Left Hippocampus | -0.010 | 0.014 | -0.690 | 0.490 | 0.927 |
|  | Right Hippocampus | -0.013 | 0.014 | -0.910 | 0.363 | 0.927 |
|  | WMH | -0.016 | 0.013 | -1.172 | 0.241 | 0.927 |
| **Bilateral Oophorectomy**  *** APOE ε4** | GM BAG | -0.001 | 0.012 | -0.069 | 0.945 | 0.945 |
|  | WM BAG | 0.019 | 0.012 | 1.639 | 0.101 | 0.927 |
|  | Left Hippocampus | 0.006 | 0.011 | 0.581 | 0.561 | 0.927 |
|  | Right Hippocampus | 0.005 | 0.011 | 0.493 | 0.622 | 0.927 |
|  | WMH | 0.013 | 0.010 | 1.239 | 0.216 | 0.927 |
| **Hysterectomy * APOE ε4** | GM BAG | -0.004 | 0.014 | -0.291 | 0.771 | 0.927 |
|  | WM BAG | -0.001 | 0.014 | -0.074 | 0.941 | 0.945 |
|  | Left Hippocampus | -0.004 | 0.013 | -0.310 | 0.756 | 0.927 |
|  | Right Hippocampus | -0.014 | 0.013 | -1.043 | 0.297 | 0.927 |
|  | WMH | -0.025 | 0.012 | -1.995 | **0.046** | 0.927 |

Significant results are highlighted in bold. False discovery rate (FDR) correction was applied across all brain measures and MHT variables listed in this table. Abbreviations: APOE = apolipoprotein, MRI = magnetic resonance imaging, S.E. = standard error, GM = grey matter, BAG = brain age gap, WM = white matter, WMH = white matter hyperintensity.
