# Supplementary material for: Menopausal hormone therapy and the female brain: Leveraging neuroimaging and prescription registry data from the UK Biobank cohort
Source: eLife. 2025 May 29;13:RP99538. doi: 10.7554/eLife.99538 (PMC12122002; doi:10.7554/eLife.99538)
Supplement: Supplementary file 8. [file elife-99538-supp8.docx]

**Supplemental File 8| Interactions between APOE ε4 genotype and menopausal hormone therapy (MHT)-related variables on brain measures in the prescription sample.**

| **MHT Variable** | **MRI Measure** | **beta** | **S.E.** | **t-value** | **p-value** | **pFDR-value** |
| --- | --- | --- | --- | --- | --- | --- |
| **MHT formulation * APOE ε4** |  |  |  |  |  |  |
| Estrogens-only | GM BAG | 0.072 | 0.067 | 1.076 | 0.282 | 0.649 |
|  | WM BAG | 0.077 | 0.067 | 1.150 | 0.250 | 0.649 |
|  | Left Hippocampus | -0.010 | 0.063 | -0.152 | 0.879 | 0.976 |
|  | Right Hippocampus | -0.095 | 0.063 | -1.501 | 0.133 | 0.649 |
|  | WMH | -0.016 | 0.060 | -0.273 | 0.785 | 0.941 |
| Estrogens+Progestin | GM BAG | -0.032 | 0.060 | -0.535 | 0.592 | 0.872 |
|  | WM BAG | -0.033 | 0.060 | -0.545 | 0.586 | 0.872 |
|  | Left Hippocampus | 0.008 | 0.057 | 0.143 | 0.887 | 0.976 |
|  | Right Hippocampus | -0.057 | 0.057 | -0.998 | 0.319 | 0.681 |
|  | WMH | -0.072 | 0.052 | -1.397 | 0.163 | 0.649 |
| **Route of Administration**  *** APOE ε4** |  |  |  |  |  |  |
| oral | GM BAG | -0.008 | 0.068 | -0.115 | 0.908 | 0.976 |
|  | WM BAG | 0.059 | 0.068 | 0.872 | 0.383 | 0.730 |
|  | Left Hippocampus | 0.003 | 0.064 | 0.039 | 0.969 | 0.981 |
|  | Right Hippocampus | -0.063 | 0.064 | -0.992 | 0.321 | 0.681 |
|  | WMH | -0.056 | 0.058 | -0.958 | 0.338 | 0.685 |
| transdermal | GM BAG | 0.077 | 0.132 | 0.579 | 0.563 | 0.865 |
|  | WM BAG | -0.104 | 0.132 | -0.784 | 0.433 | 0.778 |
|  | Left Hippocampus | 0.170 | 0.125 | 1.362 | 0.173 | 0.649 |
|  | Right Hippocampus | 0.039 | 0.125 | 0.309 | 0.757 | 0.941 |
|  | WMH | -0.139 | 0.114 | -1.219 | 0.223 | 0.649 |
| vaginal | GM BAG | 0.096 | 0.091 | 1.048 | 0.295 | 0.649 |
|  | WM BAG | 0.015 | 0.091 | 0.159 | 0.873 | 0.976 |
|  | Left Hippocampus | -0.004 | 0.086 | -0.043 | 0.965 | 0.981 |
|  | Right Hippocampus | -0.109 | 0.086 | -1.267 | 0.205 | 0.649 |
|  | WMH | 0.002 | 0.083 | 0.023 | 0.981 | 0.981 |
| injection | GM BAG | 0.558 | 0.326 | 1.712 | 0.087 | 0.649 |
|  | WM BAG | 0.106 | 0.326 | 0.327 | 0.744 | 0.941 |
|  | Left Hippocampus | -0.703 | 0.307 | -2.289 | **0.022** | 0.449 |
|  | Right Hippocampus | -0.462 | 0.307 | -1.503 | 0.133 | 0.649 |
|  | WMH | -0.114 | 0.280 | -0.408 | 0.683 | 0.934 |
| mixed | GM BAG | -0.181 | 0.104 | -1.736 | 0.083 | 0.649 |
|  | WM BAG | -0.067 | 0.104 | -0.639 | 0.523 | 0.837 |
|  | Left Hippocampus | -0.027 | 0.098 | -0.278 | 0.781 | 0.941 |
|  | Right Hippocampus | -0.114 | 0.098 | -1.162 | 0.245 | 0.649 |
|  | WMH | -0.067 | 0.090 | -0.743 | 0.458 | 0.796 |
| **Estrogen-only Forms**  *** APOE ε4** |  |  |  |  |  |  |
| Bioidentical | GM BAG | 0.092 | 0.075 | 1.229 | 0.219 | 0.649 |
|  | WM BAG | 0.032 | 0.075 | 0.422 | 0.673 | 0.928 |
|  | Left Hippocampus | 0.038 | 0.071 | 0.536 | 0.592 | 0.872 |
|  | Right Hippocampus | -0.080 | 0.071 | -1.133 | 0.257 | 0.649 |
|  | WMH | -0.040 | 0.067 | -0.601 | 0.548 | 0.857 |
| Synthetic | GM BAG | -0.223 | 0.197 | -1.132 | 0.258 | 0.649 |
|  | WM BAG | 0.271 | 0.197 | 1.373 | 0.170 | 0.649 |
|  | Left Hippocampus | -0.149 | 0.186 | -0.803 | 0.422 | 0.767 |
|  | Right Hippocampus | -0.012 | 0.186 | -0.064 | 0.949 | 0.981 |
|  | WMH | -0.155 | 0.169 | -0.916 | 0.360 | 0.693 |
| **Estrogen-only,**  **active ingredient * APOE ε4** |  |  |  |  |  |  |
| estradiol | GM BAG | 0.079 | 0.181 | 0.435 | 0.664 | 0.927 |
|  | WM BAG | -0.126 | 0.181 | -0.698 | 0.485 | 0.824 |
|  | Left Hippocampus | 0.113 | 0.170 | 0.663 | 0.508 | 0.831 |
|  | Right Hippocampus | 0.043 | 0.171 | 0.250 | 0.803 | 0.941 |
|  | WMH | -0.148 | 0.156 | -0.947 | 0.344 | 0.687 |
| estradiol hemihydrate | GM BAG | 0.087 | 0.083 | 1.052 | 0.293 | 0.649 |
|  | WM BAG | 0.057 | 0.083 | 0.692 | 0.489 | 0.824 |
|  | Left Hippocampus | 0.022 | 0.078 | 0.280 | 0.779 | 0.941 |
|  | Right Hippocampus | -0.105 | 0.078 | -1.352 | 0.177 | 0.649 |
|  | WMH | -0.018 | 0.075 | -0.240 | 0.811 | 0.941 |
| estradiol valerate | GM BAG | -0.228 | 0.516 | -0.443 | 0.658 | 0.927 |
|  | WM BAG | 0.985 | 0.516 | 1.911 | 0.056 | 0.561 |
|  | Left Hippocampus | -0.818 | 0.486 | -1.684 | 0.092 | 0.649 |
|  | Right Hippocampus | -0.537 | 0.486 | -1.104 | 0.270 | 0.649 |
|  | WMH | -0.155 | 0.443 | -0.350 | 0.726 | 0.941 |
| CEE | GM BAG | -0.332 | 0.227 | -1.465 | 0.143 | 0.649 |
|  | WM BAG | 0.120 | 0.227 | 0.530 | 0.596 | 0.872 |
|  | Left Hippocampus | -0.057 | 0.214 | -0.267 | 0.789 | 0.941 |
|  | Right Hippocampus | 0.010 | 0.214 | 0.047 | 0.963 | 0.981 |
|  | WMH | -0.191 | 0.195 | -0.979 | 0.328 | 0.681 |
| Mixed | GM BAG | 0.234 | 0.243 | 0.962 | 0.336 | 0.685 |
|  | WM BAG | 0.272 | 0.243 | 1.121 | 0.262 | 0.649 |
|  | Left Hippocampus | -0.327 | 0.229 | -1.428 | 0.153 | 0.649 |
|  | Right Hippocampus | -0.491 | 0.229 | -2.145 | **0.032** | 0.511 |
|  | WMH | 0.400 | 0.209 | 1.911 | 0.056 | 0.561 |
| **Estrogens-only,**  **Dosage (mg) * APOE ε4** |  |  |  |  |  |  |
|  | GM BAG | -0.019 | 0.072 | -0.266 | 0.791 | 0.941 |
|  | WM BAG | 0.078 | 0.072 | 1.091 | 0.276 | 0.649 |
|  | Left Hippocampus | -0.073 | 0.068 | -1.064 | 0.288 | 0.649 |
|  | Right Hippocampus | -0.003 | 0.068 | -0.045 | 0.964 | 0.981 |
|  | WMH | -0.010 | 0.059 | -0.175 | 0.862 | 0.976 |
| **Estrogens-only,**  **Duration of Use (weeks)**  *** APOE ε4** |  |  |  |  |  |  |
|  | GM BAG | -0.046 | 0.077 | -0.596 | 0.552 | 0.857 |
|  | WM BAG | 0.019 | 0.080 | 0.242 | 0.809 | 0.941 |
|  | Left Hippocampus | -0.052 | 0.078 | -0.661 | 0.510 | 0.831 |
|  | Right Hippocampus | -0.002 | 0.074 | -0.024 | 0.981 | 0.981 |
|  | WMH | -0.010 | 0.062 | -0.165 | 0.869 | 0.976 |
| **Estrogens + Progestins Form**  *** APOE ε4** |  |  |  |  |  |  |
| Bioidentical | GM BAG | -0.067 | 0.257 | -0.263 | 0.793 | 0.941 |
|  | WM BAG | -0.327 | 0.258 | -1.268 | 0.205 | 0.649 |
|  | Left Hippocampus | -0.349 | 0.243 | -1.437 | 0.151 | 0.649 |
|  | Right Hippocampus | -0.341 | 0.243 | -1.402 | 0.161 | 0.649 |
|  | WMH | 0.303 | 0.221 | 1.370 | 0.171 | 0.649 |
| Synthetic | GM BAG | -0.512 | 0.220 | -2.330 | **0.020** | 0.449 |
|  | WM BAG | -0.323 | 0.220 | -1.464 | 0.143 | 0.649 |
|  | Left Hippocampus | 0.502 | 0.207 | 2.420 | **0.016** | 0.449 |
|  | Right Hippocampus | 0.405 | 0.208 | 1.947 | 0.052 | 0.561 |
|  | WMH | -0.412 | 0.189 | -2.180 | **0.029** | 0.511 |
| Bioidentical & Synthetic | GM BAG | 0.106 | 0.114 | 0.931 | 0.352 | 0.693 |
|  | WM BAG | 0.096 | 0.114 | 0.840 | 0.401 | 0.746 |
|  | Left Hippocampus | -0.135 | 0.108 | -1.251 | 0.211 | 0.649 |
|  | Right Hippocampus | -0.292 | 0.108 | -2.709 | **0.007** | 0.449 |
|  | WMH | -0.061 | 0.098 | -0.621 | 0.535 | 0.847 |
| **Estrogens + Progestins,**  **active ingredient * APOE ε4** |  |  |  |  |  |  |
| estradiol hemihydrate &  norethisterone acetate | GM BAG | 0.247 | 0.148 | 1.662 | 0.097 | 0.649 |
|  | WM BAG | 0.137 | 0.149 | 0.925 | 0.355 | 0.693 |
|  | Left Hippocampus | -0.219 | 0.140 | -1.565 | 0.118 | 0.649 |
|  | Right Hippocampus | -0.320 | 0.140 | -2.283 | **0.022** | 0.449 |
|  | WMH | -0.209 | 0.128 | -1.635 | 0.102 | 0.649 |
| estradiol hemihydrate &  dydrogesterone | GM BAG | -0.020 | 0.264 | -0.076 | 0.940 | 0.981 |
|  | WM BAG | -0.279 | 0.264 | -1.056 | 0.291 | 0.649 |
|  | Left Hippocampus | -0.322 | 0.249 | -1.296 | 0.195 | 0.649 |
|  | Right Hippocampus | -0.349 | 0.249 | -1.400 | 0.162 | 0.649 |
|  | WMH | 0.345 | 0.226 | 1.525 | 0.127 | 0.649 |
| estradiol hemihydrate &  norethisterone | GM BAG | -0.382 | 0.293 | -1.303 | 0.193 | 0.649 |
|  | WM BAG | -0.154 | 0.294 | -0.525 | 0.599 | 0.872 |
|  | Left Hippocampus | 0.030 | 0.277 | 0.109 | 0.914 | 0.976 |
|  | Right Hippocampus | -0.324 | 0.277 | -1.169 | 0.242 | 0.649 |
|  | WMH | 0.124 | 0.252 | 0.492 | 0.622 | 0.889 |
| CEE & norgestrel | GM BAG | -0.513 | 0.253 | -2.029 | **0.042** | 0.561 |
|  | WM BAG | -0.348 | 0.253 | -1.373 | 0.170 | 0.649 |
|  | Left Hippocampus | 0.414 | 0.238 | 1.737 | 0.082 | 0.649 |
|  | Right Hippocampus | 0.437 | 0.239 | 1.830 | 0.067 | 0.633 |
|  | WMH | -0.499 | 0.217 | -2.299 | **0.022** | 0.449 |
| CEE &  medroxyprogesterone acetate | GM BAG | -0.512 | 0.470 | -1.090 | 0.276 | 0.649 |
|  | WM BAG | -0.050 | 0.470 | -0.106 | 0.915 | 0.976 |
|  | Left Hippocampus | 0.860 | 0.443 | 1.941 | 0.052 | 0.561 |
|  | Right Hippocampus | 0.493 | 0.444 | 1.111 | 0.266 | 0.649 |
|  | WMH | -0.159 | 0.403 | -0.394 | 0.693 | 0.939 |
| tibolone | GM BAG | -0.709 | 0.464 | -1.527 | 0.127 | 0.649 |
|  | WM BAG | -0.401 | 0.465 | -0.862 | 0.389 | 0.731 |
|  | Left Hippocampus | 0.881 | 0.437 | 2.013 | **0.044** | 0.561 |
|  | Right Hippocampus | 0.468 | 0.438 | 1.068 | 0.285 | 0.649 |
|  | WMH | -0.418 | 0.400 | -1.045 | 0.296 | 0.649 |
| Mixed | GM BAG | -0.039 | 0.079 | -0.493 | 0.622 | 0.889 |
|  | WM BAG | -0.026 | 0.079 | -0.328 | 0.743 | 0.941 |
|  | Left Hippocampus | 0.024 | 0.075 | 0.324 | 0.746 | 0.941 |
|  | Right Hippocampus | 0.018 | 0.075 | 0.238 | 0.812 | 0.941 |
|  | WMH | -0.057 | 0.068 | -0.831 | 0.406 | 0.746 |
| **Estrogens + Progestins,**  **Progestin Generation**  *** APOE ε4** |  |  |  |  |  |  |
| 1stGen | GM BAG | 0.113 | 0.103 | 1.096 | 0.273 | 0.649 |
|  | WM BAG | 0.040 | 0.103 | 0.387 | 0.699 | 0.939 |
|  | Left Hippocampus | -0.134 | 0.097 | -1.374 | 0.169 | 0.649 |
|  | Right Hippocampus | -0.231 | 0.097 | -2.370 | **0.018** | 0.449 |
|  | WMH | -0.104 | 0.089 | -1.162 | 0.245 | 0.649 |
| 2ndGen | GM BAG | -0.239 | 0.169 | -1.413 | 0.158 | 0.649 |
|  | WM BAG | -0.250 | 0.169 | -1.476 | 0.140 | 0.649 |
|  | Left Hippocampus | 0.024 | 0.159 | 0.148 | 0.882 | 0.976 |
|  | Right Hippocampus | 0.044 | 0.160 | 0.275 | 0.783 | 0.941 |
|  | WMH | -0.113 | 0.145 | -0.775 | 0.438 | 0.779 |
| **Estrogens + Progestins,**  **Dosage (mg) * APOE ε4** |  |  |  |  |  |  |
| Estrogens | GM BAG | 0.070 | 0.061 | 1.148 | 0.252 | 0.649 |
|  | WM BAG | 0.076 | 0.061 | 1.248 | 0.213 | 0.649 |
|  | Left Hippocampus | -0.008 | 0.059 | -0.128 | 0.899 | 0.976 |
|  | Right Hippocampus | -0.058 | 0.059 | -0.988 | 0.324 | 0.681 |
|  | WMH | -0.024 | 0.054 | -0.432 | 0.666 | 0.927 |
| Progestins | GM BAG | -0.061 | 0.221 | -0.276 | 0.783 | 0.941 |
|  | WM BAG | -0.160 | 0.222 | -0.724 | 0.470 | 0.808 |
|  | Left Hippocampus | -0.059 | 0.216 | -0.275 | 0.783 | 0.941 |
|  | Right Hippocampus | 0.113 | 0.214 | 0.528 | 0.598 | 0.872 |
|  | WMH | 0.512 | 0.197 | 2.591 | **0.010** | 0.449 |
| **Estrogens + Progestins,**  **Duration of Use (weeks)**  *** APOE ε4** |  |  |  |  |  |  |
| Estrogens | GM BAG | 0.013 | 0.070 | 0.190 | 0.850 | 0.976 |
|  | WM BAG | -0.047 | 0.072 | -0.653 | 0.514 | 0.831 |
|  | Left Hippocampus | -0.005 | 0.068 | -0.067 | 0.946 | 0.981 |
|  | Right Hippocampus | 0.043 | 0.066 | 0.655 | 0.513 | 0.831 |
|  | WMH | 0.070 | 0.064 | 1.106 | 0.270 | 0.649 |
| Progestins | GM BAG | -0.025 | 0.080 | -0.317 | 0.751 | 0.941 |
|  | WM BAG | 0.101 | 0.081 | 1.243 | 0.215 | 0.649 |
|  | Left Hippocampus | 0.059 | 0.077 | 0.763 | 0.446 | 0.784 |
|  | Right Hippocampus | -0.007 | 0.075 | -0.095 | 0.924 | 0.979 |
|  | WMH | 0.009 | 0.072 | 0.122 | 0.903 | 0.976 |

Significant results are highlighted in bold. False discovery rate (FDR) correction was applied across all brain measures and MHT variables listed in this table. Abbreviations: APOE = apolipoprotein, MRI = magnetic resonance imaging, S.E. = standard error, GM = grey matter, BAG = brain age gap, WM = white matter, WMH = white matter hyperintensity, CEE = conjugated equine estrogen, Gen = generation.
