# Supplementary material for: Menopausal hormone therapy and the female brain: Leveraging neuroimaging and prescription registry data from the UK Biobank cohort
Source: eLife. 2025 May 29;13:RP99538. doi: 10.7554/eLife.99538 (PMC12122002; doi:10.7554/eLife.99538)
Supplement: Supplementary file 9. [file elife-99538-supp9.docx]

**Supplemental File 9| Associations between menopausal hormone therapy (MHT)-related variables and brain measures in the whole sample, excluding participants with ICD-10 diagnosis known to impact the brain.**

| **MHT Variable** | **MRI Measure** | **beta** | **S.E.** | **t-value** | **p-value** | **pFDR-value** |
| --- | --- | --- | --- | --- | --- | --- |
| MHT Status | GM BAG | 0.036 | 0.009 | 4.155 | **3.27e-05** | **0.001** |
|  | WM BAG | 0.017 | 0.009 | 1.952 | 0.051 | 0.109 |
|  | Left Hippocampus | -0.021 | 0.008 | -2.615 | **0.009** | **0.032** |
|  | Right Hippocampus | -0.012 | 0.008 | -1.462 | 0.144 | 0.257 |
|  | WMH | 0.007 | 0.007 | 0.890 | 0.374 | 0.505 |
| Current MHT use | GM BAG | 0.224 | 0.040 | 5.644 | **1.70e-08** | **8.49e-07** |
|  | WM BAG | 0.160 | 0.040 | 4.042 | **5.33e-05** | **0.001** |
|  | Left Hippocampus | -0.158 | 0.037 | -4.257 | **2.08e-05** | **0.001** |
|  | Right Hippocampus | -0.130 | 0.037 | -3.531 | **4.16e-04** | **0.003** |
|  | WMH | -0.007 | 0.034 | -0.203 | 0.839 | 0.933 |
| Past MHT use | GM BAG | 0.046 | 0.022 | 2.111 | **0.035** | 0.092 |
|  | WM BAG | 0.003 | 0.022 | 0.135 | 0.892 | 0.949 |
|  | Left Hippocampus | -0.011 | 0.020 | -0.552 | 0.581 | 0.764 |
|  | Right Hippocampus | -0.002 | 0.020 | -0.108 | 0.914 | 0.952 |
|  | WMH | 0.020 | 0.018 | 1.077 | 0.281 | 0.426 |
| Age at first MHT use | GM BAG | 0.004 | 0.016 | 0.257 | 0.798 | 0.906 |
|  | WM BAG | 0.000 | 0.016 | -0.007 | 0.995 | 0.995 |
|  | Left Hippocampus | 0.004 | 0.016 | 0.262 | 0.793 | 0.906 |
|  | Right Hippocampus | -0.006 | 0.016 | -0.405 | 0.685 | 0.857 |
|  | WMH | -0.022 | 0.014 | -1.585 | 0.113 | 0.209 |
| Age at first MHT use relative  to age at menopause | GM BAG | 0.024 | 0.017 | 1.388 | 0.165 | 0.275 |
|  | WM BAG | 0.036 | 0.017 | 2.083 | **0.037** | 0.092 |
|  | Left Hippocampus | 0.009 | 0.017 | 0.524 | 0.600 | 0.770 |
|  | Right Hippocampus | -0.017 | 0.017 | -1.018 | 0.309 | 0.441 |
|  | WMH | -0.004 | 0.015 | -0.298 | 0.765 | 0.906 |
| Age at last MHT use | GM BAG | 0.039 | 0.019 | 2.069 | **0.039** | 0.092 |
|  | WM BAG | 0.032 | 0.019 | 1.662 | 0.097 | 0.192 |
|  | Left Hippocampus | -0.030 | 0.018 | -1.646 | 0.100 | 0.192 |
|  | Right Hippocampus | -0.022 | 0.018 | -1.187 | 0.235 | 0.368 |
|  | WMH | 0.023 | 0.017 | 1.364 | 0.173 | 0.278 |
| Age at last MHT use relative  to age at menopause | GM BAG | 0.053 | 0.019 | 2.798 | **0.005** | **0.022** |
|  | WM BAG | 0.062 | 0.019 | 3.254 | **0.001** | **0.006** |
|  | Left Hippocampus | -0.039 | 0.019 | -2.089 | **0.037** | 0.092 |
|  | Right Hippocampus | -0.038 | 0.019 | -2.052 | **0.040** | 0.092 |
|  | WMH | 0.043 | 0.017 | 2.588 | **0.010** | **0.032** |
| Duration of MHT use | GM BAG | 0.064 | 0.017 | 3.787 | 1.55e-04 | **0.002** |
|  | WM BAG | 0.054 | 0.017 | 3.192 | **0.001** | **0.007** |
|  | Left Hippocampus | -0.055 | 0.016 | -3.465 | **0.001** | **0.003** |
|  | Right Hippocampus | -0.035 | 0.016 | -2.235 | **0.025** | 0.075 |
|  | WMH | 0.028 | 0.015 | 1.942 | 0.052 | 0.109 |
| Bilateral Oophorectomy | GM BAG | -0.033 | 0.013 | -2.594 | **0.009** | **0.032** |
|  | WM BAG | -0.004 | 0.013 | -0.291 | 0.771 | 0.906 |
|  | Left Hippocampus | 0.012 | 0.012 | 1.046 | 0.296 | 0.435 |
|  | Right Hippocampus | -0.001 | 0.012 | -0.076 | 0.939 | 0.958 |
|  | WMH | 0.002 | 0.011 | 0.164 | 0.869 | 0.945 |
| Hysterectomy | GM BAG | -0.047 | 0.013 | -3.497 | **4.74e-04** | **0.003** |
|  | WM BAG | -0.019 | 0.014 | -1.401 | 0.161 | 0.275 |
|  | Left Hippocampus | 0.038 | 0.013 | 2.990 | **0.003** | **0.013** |
|  | Right Hippocampus | 0.030 | 0.013 | 2.406 | **0.016** | 0.051 |
|  | WMH | -0.012 | 0.012 | -0.991 | 0.322 | 0.447 |

Significant results are highlighted in bold. False discovery rate (FDR) correction was applied across all brain measures and MHT variables listed in this table. Abbreviations: MRI = magnetic resonance imaging, S.E. = standard error, GM = grey matter, BAG = brain age gap, WM = white matter, WMH = white matter hyperintensity.
