# Supplementary material for: Menopausal hormone therapy and the female brain: Leveraging neuroimaging and prescription registry data from the UK Biobank cohort
Source: eLife. 2025 May 29;13:RP99538. doi: 10.7554/eLife.99538 (PMC12122002; doi:10.7554/eLife.99538)
Supplement: Supplementary file 10. [file elife-99538-supp10.docx]

**Supplemental File 10| Associations between menopausal hormone therapy (MHT)-related variables and brain measures in the prescription MHT sample, excluding participants with ICD-10 diagnosis known to impact the brain.**

| **MHT Variable** | **MRI Measure** | **beta** | **S.E.** | **t-value** | **p-value** | **pFDR-value** |
| --- | --- | --- | --- | --- | --- | --- |
| **MHT formulation** |  |  |  |  |  |  |
| Estrogens-only | GM BAG | -0.008 | 0.071 | -0.118 | 0.906 | 0.990 |
|  | WM BAG | 0.044 | 0.071 | 0.625 | 0.532 | 0.990 |
|  | Left Hippocampus | -0.054 | 0.067 | -0.809 | 0.419 | 0.990 |
|  | Right Hippocampus | -0.058 | 0.067 | -0.861 | 0.389 | 0.990 |
|  | WMH | 0.021 | 0.062 | 0.333 | 0.739 | 0.990 |
| Estrogens+Progestin | GM BAG | -0.045 | 0.060 | -0.739 | 0.460 | 0.990 |
|  | WM BAG | 0.080 | 0.060 | 1.332 | 0.183 | 0.990 |
|  | Left Hippocampus | 0.025 | 0.057 | 0.439 | 0.661 | 0.990 |
|  | Right Hippocampus | 0.023 | 0.057 | 0.407 | 0.684 | 0.990 |
|  | WMH | 0.007 | 0.052 | 0.138 | 0.890 | 0.990 |
| **Route of Administration** |  |  |  |  |  |  |
| oral | GM BAG | -0.081 | 0.070 | -1.163 | 0.245 | 0.990 |
|  | WM BAG | 0.079 | 0.070 | 1.138 | 0.255 | 0.990 |
|  | Left Hippocampus | 0.011 | 0.066 | 0.160 | 0.873 | 0.990 |
|  | Right Hippocampus | 0.017 | 0.066 | 0.257 | 0.797 | 0.990 |
|  | WMH | 0.103 | 0.060 | 1.711 | 0.087 | 0.990 |
| transdermal | GM BAG | -0.248 | 0.134 | -1.848 | 0.065 | 0.990 |
|  | WM BAG | -0.086 | 0.134 | -0.643 | 0.520 | 0.990 |
|  | Left Hippocampus | -0.023 | 0.126 | -0.183 | 0.855 | 0.990 |
|  | Right Hippocampus | -0.046 | 0.126 | -0.368 | 0.713 | 0.990 |
|  | WMH | -0.117 | 0.115 | -1.011 | 0.312 | 0.990 |
| vaginal | GM BAG | 0.168 | 0.099 | 1.696 | 0.090 | 0.990 |
|  | WM BAG | 0.146 | 0.099 | 1.470 | 0.142 | 0.990 |
|  | Left Hippocampus | -0.036 | 0.093 | -0.389 | 0.697 | 0.990 |
|  | Right Hippocampus | -0.023 | 0.093 | -0.251 | 0.802 | 0.990 |
|  | WMH | 0.092 | 0.088 | 1.050 | 0.294 | 0.990 |
| injection | GM BAG | 0.536 | 0.353 | 1.515 | 0.130 | 0.990 |
|  | WM BAG | 0.290 | 0.354 | 0.820 | 0.412 | 0.990 |
|  | Left Hippocampus | -0.173 | 0.333 | -0.518 | 0.605 | 0.990 |
|  | Right Hippocampus | 0.182 | 0.333 | 0.547 | 0.584 | 0.990 |
|  | WMH | -0.054 | 0.302 | -0.181 | 0.857 | 0.990 |
| mixed | GM BAG | -0.046 | 0.096 | -0.481 | 0.630 | 0.990 |
|  | WM BAG | 0.024 | 0.096 | 0.249 | 0.804 | 0.990 |
|  | Left Hippocampus | 0.003 | 0.090 | 0.031 | 0.976 | 0.990 |
|  | Right Hippocampus | -0.047 | 0.090 | -0.516 | 0.606 | 0.990 |
|  | WMH | -0.161 | 0.083 | -1.940 | 0.052 | 0.990 |
| **Estrogen-only Forms** |  |  |  |  |  |  |
| Bioidentical | GM BAG | 0.012 | 0.080 | 0.151 | 0.880 | 0.990 |
|  | WM BAG | 0.048 | 0.080 | 0.602 | 0.547 | 0.990 |
|  | Left Hippocampus | -0.039 | 0.075 | -0.518 | 0.604 | 0.990 |
|  | Right Hippocampus | -0.031 | 0.075 | -0.417 | 0.677 | 0.990 |
|  | WMH | 0.008 | 0.070 | 0.117 | 0.907 | 0.990 |
| Synthetic | GM BAG | 0.146 | 0.230 | 0.636 | 0.525 | 0.990 |
|  | WM BAG | 0.105 | 0.230 | 0.455 | 0.649 | 0.990 |
|  | Left Hippocampus | -0.100 | 0.217 | -0.462 | 0.644 | 0.990 |
|  | Right Hippocampus | -0.005 | 0.216 | -0.025 | 0.980 | 0.990 |
|  | WMH | 0.063 | 0.196 | 0.319 | 0.750 | 0.990 |
| **Estrogen-only,**  **active ingredient** |  |  |  |  |  |  |
| estradiol | GM BAG | -0.243 | 0.186 | -1.306 | 0.192 | 0.990 |
|  | WM BAG | -0.174 | 0.186 | -0.935 | 0.350 | 0.990 |
|  | Left Hippocampus | -0.108 | 0.175 | -0.614 | 0.539 | 0.990 |
|  | Right Hippocampus | -0.009 | 0.175 | -0.049 | 0.961 | 0.990 |
|  | WMH | -0.123 | 0.162 | -0.759 | 0.448 | 0.990 |
| estradiol hemihydrate | GM BAG | 0.069 | 0.088 | 0.779 | 0.436 | 0.990 |
|  | WM BAG | 0.098 | 0.088 | 1.109 | 0.268 | 0.990 |
|  | Left Hippocampus | -0.024 | 0.083 | -0.284 | 0.776 | 0.990 |
|  | Right Hippocampus | -0.036 | 0.083 | -0.438 | 0.661 | 0.990 |
|  | WMH | 0.038 | 0.078 | 0.487 | 0.626 | 0.990 |
| estradiol valerate | GM BAG | 0.482 | 0.500 | 0.965 | 0.335 | 0.990 |
|  | WM BAG | 0.258 | 0.500 | 0.515 | 0.606 | 0.990 |
|  | Left Hippocampus | -0.030 | 0.471 | -0.064 | 0.949 | 0.990 |
|  | Right Hippocampus | 0.386 | 0.470 | 0.822 | 0.411 | 0.990 |
|  | WMH | 0.203 | 0.426 | 0.476 | 0.634 | 0.990 |
| CEE | GM BAG | 0.056 | 0.259 | 0.215 | 0.829 | 0.990 |
|  | WM BAG | 0.064 | 0.259 | 0.247 | 0.805 | 0.990 |
|  | Left Hippocampus | -0.118 | 0.244 | -0.486 | 0.627 | 0.990 |
|  | Right Hippocampus | -0.110 | 0.243 | -0.450 | 0.653 | 0.990 |
|  | WMH | 0.024 | 0.221 | 0.110 | 0.912 | 0.990 |
| Mixed | GM BAG | -0.255 | 0.197 | -1.294 | 0.196 | 0.990 |
|  | WM BAG | -0.027 | 0.197 | -0.136 | 0.892 | 0.990 |
|  | Left Hippocampus | -0.114 | 0.185 | -0.616 | 0.538 | 0.990 |
|  | Right Hippocampus | -0.255 | 0.185 | -1.374 | 0.169 | 0.990 |
|  | WMH | 0.058 | 0.168 | 0.348 | 0.728 | 0.990 |
| **Estrogens-only,**  **Dosage (mg)** |  |  |  |  |  |  |
|  | GM BAG | -0.047 | 0.078 | -0.603 | 0.547 | 0.990 |
|  | WM BAG | -0.097 | 0.079 | -1.232 | 0.219 | 0.990 |
|  | Left Hippocampus | -0.031 | 0.075 | -0.411 | 0.681 | 0.990 |
|  | Right Hippocampus | -0.041 | 0.074 | -0.550 | 0.583 | 0.990 |
|  | WMH | -0.026 | 0.064 | -0.399 | 0.690 | 0.990 |
| **Estrogens-only,**  **Duration of Use (weeks)** |  |  |  |  |  |  |
|  | GM BAG | -0.101 | 0.086 | -1.171 | 0.244 | 0.990 |
|  | WM BAG | -0.131 | 0.092 | -1.431 | 0.155 | 0.990 |
|  | Left Hippocampus | -0.003 | 0.089 | -0.033 | 0.974 | 0.990 |
|  | Right Hippocampus | 0.003 | 0.084 | 0.041 | 0.968 | 0.990 |
|  | WMH | -0.158 | 0.070 | -2.248 | **0.026** | 0.990 |
| **Estrogens + Progestins Form** |  |  |  |  |  |  |
| Bioidentical | GM BAG | -0.248 | 0.258 | -0.962 | 0.336 | 0.990 |
|  | WM BAG | 0.000 | 0.259 | 0.000 | 1.000 | 1.000 |
|  | Left Hippocampus | 0.091 | 0.243 | 0.372 | 0.710 | 0.990 |
|  | Right Hippocampus | 0.388 | 0.243 | 1.595 | 0.111 | 0.990 |
|  | WMH | -0.011 | 0.221 | -0.049 | 0.961 | 0.990 |
| Synthetic | GM BAG | -0.099 | 0.196 | -0.506 | 0.613 | 0.990 |
|  | WM BAG | 0.133 | 0.197 | 0.678 | 0.498 | 0.990 |
|  | Left Hippocampus | 0.182 | 0.185 | 0.980 | 0.327 | 0.990 |
|  | Right Hippocampus | 0.153 | 0.185 | 0.826 | 0.409 | 0.990 |
|  | WMH | 0.170 | 0.168 | 1.012 | 0.312 | 0.990 |
| Bioidentical & Synthetic | GM BAG | -0.063 | 0.111 | -0.564 | 0.573 | 0.990 |
|  | WM BAG | -0.026 | 0.112 | -0.236 | 0.813 | 0.990 |
|  | Left Hippocampus | -0.049 | 0.105 | -0.463 | 0.644 | 0.990 |
|  | Right Hippocampus | -0.071 | 0.105 | -0.674 | 0.500 | 0.990 |
|  | WMH | 0.002 | 0.096 | 0.021 | 0.983 | 0.990 |
| **Estrogens + Progestins,**  **active ingredient** |  |  |  |  |  |  |
| estradiol hemihydrate &  norethisterone acetate | GM BAG | -0.176 | 0.151 | -1.167 | 0.243 | 0.990 |
|  | WM BAG | -0.103 | 0.151 | -0.680 | 0.497 | 0.990 |
|  | Left Hippocampus | 0.147 | 0.142 | 1.034 | 0.301 | 0.990 |
|  | Right Hippocampus | 0.078 | 0.142 | 0.547 | 0.585 | 0.990 |
|  | WMH | -0.011 | 0.130 | -0.086 | 0.932 | 0.990 |
| estradiol hemihydrate &  dydrogesterone | GM BAG | -0.355 | 0.277 | -1.283 | 0.200 | 0.990 |
|  | WM BAG | -0.114 | 0.277 | -0.412 | 0.680 | 0.990 |
|  | Left Hippocampus | 0.012 | 0.261 | 0.047 | 0.962 | 0.990 |
|  | Right Hippocampus | 0.380 | 0.261 | 1.455 | 0.146 | 0.990 |
|  | WMH | -0.068 | 0.237 | -0.286 | 0.775 | 0.990 |
| estradiol hemihydrate &  norethisterone | GM BAG | 0.276 | 0.301 | 0.916 | 0.360 | 0.990 |
|  | WM BAG | -0.153 | 0.302 | -0.509 | 0.611 | 0.990 |
|  | Left Hippocampus | -0.332 | 0.284 | -1.168 | 0.243 | 0.990 |
|  | Right Hippocampus | -0.345 | 0.284 | -1.216 | 0.224 | 0.990 |
|  | WMH | 0.147 | 0.257 | 0.570 | 0.569 | 0.990 |
| CEE & norgestrel | GM BAG | -0.077 | 0.229 | -0.336 | 0.737 | 0.990 |
|  | WM BAG | -0.029 | 0.230 | -0.127 | 0.899 | 0.990 |
|  | Left Hippocampus | 0.129 | 0.217 | 0.597 | 0.551 | 0.990 |
|  | Right Hippocampus | 0.033 | 0.216 | 0.154 | 0.878 | 0.990 |
|  | WMH | 0.146 | 0.196 | 0.743 | 0.458 | 0.990 |
| CEE &  medroxyprogesterone acetate | GM BAG | -0.159 | 0.377 | -0.421 | 0.674 | 0.990 |
|  | WM BAG | 0.566 | 0.378 | 1.497 | 0.134 | 0.990 |
|  | Left Hippocampus | 0.319 | 0.356 | 0.896 | 0.370 | 0.990 |
|  | Right Hippocampus | 0.475 | 0.355 | 1.337 | 0.181 | 0.990 |
|  | WMH | 0.231 | 0.322 | 0.718 | 0.473 | 0.990 |
| tibolone | GM BAG | -0.444 | 0.277 | -1.603 | 0.109 | 0.990 |
|  | WM BAG | 0.020 | 0.278 | 0.072 | 0.943 | 0.990 |
|  | Left Hippocampus | 0.028 | 0.262 | 0.109 | 0.913 | 0.990 |
|  | Right Hippocampus | 0.166 | 0.261 | 0.635 | 0.525 | 0.990 |
|  | WMH | -0.173 | 0.246 | -0.701 | 0.483 | 0.990 |
| Mixed | GM BAG | 0.043 | 0.083 | 0.522 | 0.602 | 0.990 |
|  | WM BAG | 0.151 | 0.083 | 1.819 | 0.069 | 0.990 |
|  | Left Hippocampus | 0.025 | 0.078 | 0.321 | 0.748 | 0.990 |
|  | Right Hippocampus | -0.005 | 0.078 | -0.068 | 0.946 | 0.990 |
|  | WMH | 0.009 | 0.072 | 0.129 | 0.898 | 0.990 |
| **Estrogens + Progestins,**  **Progestin Generation** |  |  |  |  |  |  |
| 1stGen | GM BAG | -0.032 | 0.101 | -0.315 | 0.753 | 0.990 |
|  | WM BAG | 0.053 | 0.101 | 0.529 | 0.597 | 0.990 |
|  | Left Hippocampus | 0.003 | 0.095 | 0.030 | 0.976 | 0.990 |
|  | Right Hippocampus | 0.008 | 0.095 | 0.086 | 0.932 | 0.990 |
|  | WMH | 0.003 | 0.087 | 0.035 | 0.972 | 0.990 |
| 2ndGen | GM BAG | -0.071 | 0.160 | -0.440 | 0.660 | 0.990 |
|  | WM BAG | 0.022 | 0.161 | 0.136 | 0.892 | 0.990 |
|  | Left Hippocampus | 0.055 | 0.151 | 0.362 | 0.717 | 0.990 |
|  | Right Hippocampus | 0.101 | 0.151 | 0.669 | 0.504 | 0.990 |
|  | WMH | 0.086 | 0.137 | 0.624 | 0.532 | 0.990 |
| **Estrogens + Progestins,**  **Dosage (mg)** |  |  |  |  |  |  |
| Estrogens | GM BAG | -0.098 | 0.060 | -1.625 | 0.105 | 0.990 |
|  | WM BAG | 0.009 | 0.061 | 0.149 | 0.882 | 0.990 |
|  | Left Hippocampus | -0.017 | 0.059 | -0.286 | 0.775 | 0.990 |
|  | Right Hippocampus | 0.045 | 0.059 | 0.767 | 0.443 | 0.990 |
|  | WMH | 0.058 | 0.055 | 1.054 | 0.293 | 0.990 |
| Progestins | GM BAG | 0.041 | 0.060 | 0.686 | 0.493 | 0.990 |
|  | WM BAG | 0.040 | 0.060 | 0.671 | 0.503 | 0.990 |
|  | Left Hippocampus | 0.079 | 0.058 | 1.368 | 0.173 | 0.990 |
|  | Right Hippocampus | 0.082 | 0.058 | 1.427 | 0.155 | 0.990 |
|  | WMH | 0.057 | 0.053 | 1.088 | 0.278 | 0.990 |
| **Estrogens + Progestins,**  **Duration of Use (weeks)** | |  |  |  |  |  |
| Estrogens | GM BAG | 0.091 | 0.074 | 1.232 | 0.219 | 0.990 |
|  | WM BAG | -0.010 | 0.076 | -0.129 | 0.898 | 0.990 |
|  | Left Hippocampus | -0.172 | 0.071 | -2.405 | **0.017** | 0.990 |
|  | Right Hippocampus | -0.177 | 0.070 | -2.522 | **0.012** | 0.990 |
|  | WMH | -0.089 | 0.067 | -1.317 | 0.189 | 0.990 |
| Progestins | GM BAG | -0.003 | 0.078 | -0.034 | 0.973 | 0.990 |
|  | WM BAG | 0.062 | 0.080 | 0.775 | 0.439 | 0.990 |
|  | Left Hippocampus | -0.017 | 0.075 | -0.221 | 0.826 | 0.990 |
|  | Right Hippocampus | 0.030 | 0.074 | 0.407 | 0.684 | 0.990 |
|  | WMH | 0.023 | 0.071 | 0.330 | 0.742 | 0.990 |

Significant results are highlighted in bold. False discovery rate (FDR) correction was applied across all brain measures and MHT variables listed in this table. Abbreviations: MRI = magnetic resonance imaging, S.E. = standard error, GM = grey matter, BAG = brain age gap, WM = white matter, WMH = white matter hyperintensity, CEE = conjugated equine estrogen, Gen = generation.
