# Supplementary material for: Menopausal hormone therapy and the female brain: Leveraging neuroimaging and prescription registry data from the UK Biobank cohort
Source: eLife. 2025 May 29;13:RP99538. doi: 10.7554/eLife.99538 (PMC12122002; doi:10.7554/eLife.99538)
Supplement: Supplementary file 11. [file elife-99538-supp11.docx]

**Supplemental File 11| Associations between menopausal hormone therapy (MHT)-related variables and brain measures in the whole sample, also adjusting for age^2^.**

| **MHT Variable** | **MRI Measure** | **beta** | **S.E.** | **t-value** | **p-value** | **pFDR-value** |
| --- | --- | --- | --- | --- | --- | --- |
| MHT Status | BAG GM | 0.029 | 0.008 | 3.513 | **4.44e-04** | **0.003** |
|  | BAG WM | 0.015 | 0.008 | 1.844 | 0.065 | 0.136 |
|  | Left Hippocampus | -0.017 | 0.008 | -2.236 | **0.025** | 0.064 |
|  | Right Hippocampus | -0.007 | 0.008 | -0.921 | 0.357 | 0.497 |
|  | WMH | 0.007 | 0.007 | 0.918 | 0.359 | 0.497 |
| Current MHT use | BAG GM | 0.217 | 0.037 | 5.798 | **6.84e-09** | **3.42e-07** |
|  | BAG WM | 0.154 | 0.038 | 4.099 | **4.18e-05** | **4.18e-04** |
|  | Left Hippocampus | -0.152 | 0.035 | -4.349 | **1.38e-05** | **1.72e-04** |
|  | Right Hippocampus | -0.129 | 0.035 | -3.678 | **2.36e-04** | **0.002** |
|  | WMH | -0.005 | 0.032 | -0.161 | 0.872 | 0.948 |
| Past MHT use | BAG GM | 0.026 | 0.021 | 1.268 | 0.205 | 0.353 |
|  | BAG WM | 0.001 | 0.021 | 0.069 | 0.945 | 0.982 |
|  | Left Hippocampus | -0.001 | 0.019 | -0.048 | 0.962 | 0.982 |
|  | Right Hippocampus | 0.014 | 0.019 | 0.715 | 0.475 | 0.597 |
|  | WMH | 0.019 | 0.018 | 1.048 | 0.295 | 0.460 |
| Age at first MHT use | BAG GM | 0.003 | 0.016 | 0.213 | 0.832 | 0.945 |
|  | BAG WM | 0.000 | 0.015 | -0.018 | 0.986 | 0.986 |
|  | Left Hippocampus | 0.010 | 0.015 | 0.685 | 0.493 | 0.602 |
|  | Right Hippocampus | -0.001 | 0.015 | -0.055 | 0.956 | 0.982 |
|  | WMH | -0.024 | 0.013 | -1.792 | 0.073 | 0.147 |
| Age at first MHT use relative  to age at menopause | BAG GM | 0.018 | 0.016 | 1.092 | 0.275 | 0.443 |
|  | BAG WM | 0.023 | 0.016 | 1.388 | 0.165 | 0.306 |
|  | Left Hippocampus | 0.013 | 0.016 | 0.795 | 0.427 | 0.562 |
|  | Right Hippocampus | -0.016 | 0.016 | -1.027 | 0.304 | 0.461 |
|  | WMH | -0.010 | 0.014 | -0.711 | 0.477 | 0.597 |
| Age at last MHT use | BAG GM | 0.044 | 0.018 | 2.448 | **0.014** | **0.042** |
|  | BAG WM | 0.037 | 0.018 | 2.075 | **0.038** | 0.091 |
|  | Left Hippocampus | -0.033 | 0.017 | -1.901 | 0.057 | 0.125 |
|  | Right Hippocampus | -0.024 | 0.017 | -1.408 | 0.159 | 0.306 |
|  | WMH | 0.018 | 0.016 | 1.155 | 0.248 | 0.414 |
| Age at last MHT use relative  to age at menopause | BAG GM | 0.056 | 0.018 | 3.060 | **0.002** | **0.009** |
|  | BAG WM | 0.057 | 0.018 | 3.150 | **0.002** | **0.008** |
|  | Left Hippocampus | -0.045 | 0.018 | -2.564 | **0.010** | **0.035** |
|  | Right Hippocampus | -0.045 | 0.017 | -2.600 | **0.009** | **0.033** |
|  | WMH | 0.035 | 0.016 | 2.231 | **0.026** | 0.064 |
| Duration of MHT use | BAG GM | 0.074 | 0.016 | 4.601 | **4.33e-06** | **1.08e-04** |
|  | BAG WM | 0.064 | 0.016 | 3.964 | **7.51e-05** | **0.001** |
|  | Left Hippocampus | -0.067 | 0.015 | -4.474 | **7.89e-06** | **1.32e-04** |
|  | Right Hippocampus | -0.046 | 0.015 | -3.107 | **0.002** | **0.009** |
|  | WMH | 0.028 | 0.014 | 1.968 | **0.049** | 0.112 |
| Bilateral Oophorectomy | BAG GM | -0.031 | 0.013 | -2.476 | **0.013** | **0.042** |
|  | BAG WM | -0.002 | 0.013 | -0.189 | 0.850 | 0.945 |
|  | Left Hippocampus | 0.011 | 0.012 | 0.901 | 0.368 | 0.497 |
|  | Right Hippocampus | -0.003 | 0.012 | -0.256 | 0.798 | 0.945 |
|  | WMH | 0.002 | 0.011 | 0.221 | 0.825 | 0.945 |
| Hysterectomy | BAG GM | -0.046 | 0.013 | -3.409 | **0.001** | **0.004** |
|  | BAG WM | -0.018 | 0.013 | -1.325 | 0.185 | 0.331 |
|  | Left Hippocampus | 0.036 | 0.013 | 2.885 | **0.004** | **0.015** |
|  | Right Hippocampus | 0.028 | 0.012 | 2.279 | **0.023** | 0.063 |
|  | WMH | -0.011 | 0.012 | -0.951 | 0.342 | 0.497 |

Significant results are highlighted in bold. False discovery rate (FDR) correction was applied across all brain measures and MHT variables listed in this table. Abbreviations: MRI = magnetic resonance imaging, S.E. = standard error, GM = grey matter, BAG = brain age gap, WM = white matter, WMH = white matter hyperintensity.
