# Supplementary material for: Menopausal hormone therapy and the female brain: Leveraging neuroimaging and prescription registry data from the UK Biobank cohort
Source: eLife. 2025 May 29;13:RP99538. doi: 10.7554/eLife.99538 (PMC12122002; doi:10.7554/eLife.99538)
Supplement: Supplementary file 12. [file elife-99538-supp12.docx]

**Supplemental File 12| Associations between menopausal hormone therapy (MHT)-related variables and brain measures in the whole sample, after removal of extreme values.**

| **MHT Variable** | **MRI Measure** | **beta** | **S.E.** | **t-value** | **p-value** | **pFDR-value** |
| --- | --- | --- | --- | --- | --- | --- |
| Age at first MHT use | GM BAG | 0.001 | 0.016 | 0.059 | 0.953 | 0.953 |
|  | WM BAG | -0.005 | 0.016 | -0.300 | 0.764 | 0.882 |
|  | Left Hippocampus | 0.004 | 0.015 | 0.285 | 0.776 | 0.882 |
|  | Right Hippocampus | -0.010 | 0.015 | -0.659 | 0.510 | 0.637 |
|  | WMH | -0.028 | 0.014 | -2.041 | **0.041** | 0.086 |
| Age at first MHT use relative | GM BAG | 0.026 | 0.018 | 1.456 | 0.145 | 0.242 |
| to age at menopause | WM BAG | 0.041 | 0.018 | 2.333 | **0.020** | **0.049** |
|  | Left Hippocampus | 0.002 | 0.017 | 0.132 | 0.895 | 0.933 |
|  | Right Hippocampus | -0.038 | 0.017 | -2.207 | **0.027** | 0.062 |
|  | WMH | -0.002 | 0.015 | -0.164 | 0.870 | 0.933 |
| Age at last MHT use | GM BAG | 0.050 | 0.018 | 2.743 | **0.006** | **0.026** |
|  | WM BAG | 0.036 | 0.018 | 1.986 | 0.047 | 0.091 |
|  | Left Hippocampus | -0.025 | 0.018 | -1.423 | 0.155 | 0.242 |
|  | Right Hippocampus | -0.024 | 0.017 | -1.365 | 0.172 | 0.253 |
|  | WMH | 0.011 | 0.016 | 0.727 | 0.467 | 0.614 |
| Age at last MHT use relative | GM BAG | 0.076 | 0.020 | 3.892 | **1.02e-04** | **0.001** |
| to age at menopause | WM BAG | 0.079 | 0.019 | 4.042 | **5.44e-05** | **0.001** |
|  | Left Hippocampus | -0.048 | 0.019 | -2.511 | **0.012** | **0.038** |
|  | Right Hippocampus | -0.056 | 0.019 | -2.938 | **0.003** | **0.024** |
|  | WMH | 0.031 | 0.017 | 1.812 | 0.070 | 0.125 |
| Duration of MHT use | GM BAG | 0.051 | 0.018 | 2.819 | **0.005** | **0.024** |
|  | WM BAG | 0.048 | 0.018 | 2.640 | **0.008** | **0.030** |
|  | Left Hippocampus | -0.048 | 0.017 | -2.846 | **0.004** | **0.024** |
|  | Right Hippocampus | -0.022 | 0.017 | -1.316 | 0.188 | 0.262 |
|  | WMH | 0.039 | 0.016 | 2.431 | **0.015** | **0.042** |

Significant results are highlighted in bold. False discovery rate (FDR) correction was applied across all brain measures and MHT variables listed in this table. Abbreviations: MRI = magnetic resonance imaging, S.E. = standard error, GM = grey matter, BAG = brain age gap, WM = white matter, WMH = white matter hyperintensity.
