# Supplementary material for: Menopausal hormone therapy and the female brain: Leveraging neuroimaging and prescription registry data from the UK Biobank cohort
Source: eLife. 2025 May 29;13:RP99538. doi: 10.7554/eLife.99538 (PMC12122002; doi:10.7554/eLife.99538)
Supplement: Supplementary file 13. [file elife-99538-supp13.docx]

**Supplemental File 13**| **Detected extreme values of continuous menopausal hormone therapy (MHT)-related variables using the median absolute deviation method.**

|  |  |  |  | **Number of extreme values** | | |
| --- | --- | --- | --- | --- | --- | --- |
| **MHT Variable** | **Median** | **MAD** | **Limits*** | **Low** | **High** | **Total** |
| Age started MHT | 50 | 4.45 | 36.66 – 63.34 | 76 | 29 | 105 |
| Age last used MHT | 54 | 5.93 | 36.21 – 71.79 | 14 | 13 | 27 |
| Age at Menopause | 51 | 2.97 | 42.10 – 59.90 | 715 | 158 | 873 |

*Limits of acceptable range of values. Abbreviation: MHT = hormone therapy, MAD = median absolute deviation.
