# Supplementary material for: Menopausal hormone therapy and the female brain: Leveraging neuroimaging and prescription registry data from the UK Biobank cohort
Source: eLife. 2025 May 29;13:RP99538. doi: 10.7554/eLife.99538 (PMC12122002; doi:10.7554/eLife.99538)
Supplement: Supplementary file 14. [file elife-99538-supp14.docx]

**Supplemental File 14| Associations between menopausal hormone therapy (MHT)-related variables and brain measures in the prescription MHT sample, adjusting for additional covariates.**

| **MHT Variable** | **MRI Measure** | **beta** | **S.E.** | **t-value** | **p-value** | **pFDR-value** |
| --- | --- | --- | --- | --- | --- | --- |
| **MHT formulation** |  |  |  |  |  |  |
| Estrogens-only | GM BAG | -0.014 | 0.078 | -0.183 | 0.855 | 0.999 |
|  | WM BAG | 0.048 | 0.078 | 0.613 | 0.540 | 0.999 |
|  | Left Hippocampus | -0.070 | 0.073 | -0.955 | 0.339 | 0.999 |
|  | Right Hippocampus | -0.060 | 0.073 | -0.811 | 0.418 | 0.999 |
|  | WMH | 0.055 | 0.068 | 0.807 | 0.420 | 0.999 |
| Estrogens+Progestin | GM BAG | 0.011 | 0.060 | 0.182 | 0.856 | 0.999 |
|  | WM BAG | 0.106 | 0.060 | 1.768 | 0.077 | 0.999 |
|  | Left Hippocampus | 0.023 | 0.056 | 0.410 | 0.682 | 0.999 |
|  | Right Hippocampus | 0.007 | 0.056 | 0.117 | 0.907 | 0.999 |
|  | WMH | 0.045 | 0.052 | 0.875 | 0.382 | 0.999 |
| **Route of Administration** |  |  |  |  |  |  |
| oral | GM BAG | -0.060 | 0.071 | -0.856 | 0.392 | 0.999 |
|  | WM BAG | 0.074 | 0.071 | 1.041 | 0.298 | 0.999 |
|  | Left Hippocampus | -0.008 | 0.067 | -0.122 | 0.903 | 0.999 |
|  | Right Hippocampus | 0.026 | 0.067 | 0.396 | 0.692 | 0.999 |
|  | WMH | 0.121 | 0.061 | 1.984 | **0.047** | 0.986 |
| transdermal | GM BAG | -0.208 | 0.142 | -1.469 | 0.142 | 0.999 |
|  | WM BAG | 0.033 | 0.142 | 0.229 | 0.819 | 0.999 |
|  | Left Hippocampus | -0.029 | 0.134 | -0.215 | 0.830 | 0.999 |
|  | Right Hippocampus | -0.100 | 0.134 | -0.747 | 0.455 | 0.999 |
|  | WMH | -0.099 | 0.125 | -0.794 | 0.427 | 0.999 |
| vaginal | GM BAG | 0.097 | 0.103 | 0.941 | 0.347 | 0.999 |
|  | WM BAG | 0.067 | 0.103 | 0.652 | 0.515 | 0.999 |
|  | Left Hippocampus | -0.001 | 0.097 | -0.014 | 0.989 | 0.999 |
|  | Right Hippocampus | 0.038 | 0.098 | 0.393 | 0.694 | 0.999 |
|  | WMH | 0.072 | 0.091 | 0.782 | 0.434 | 0.999 |
| injection | GM BAG | 0.993 | 0.406 | 2.444 | **0.015** | 0.847 |
|  | WM BAG | 0.374 | 0.408 | 0.917 | 0.359 | 0.999 |
|  | Left Hippocampus | -0.660 | 0.384 | -1.717 | 0.086 | 0.999 |
|  | Right Hippocampus | -0.396 | 0.385 | -1.029 | 0.303 | 0.999 |
|  | WMH | -0.208 | 0.347 | -0.601 | 0.548 | 0.999 |
| mixed | GM BAG | 0.078 | 0.097 | 0.800 | 0.424 | 0.999 |
|  | WM BAG | 0.128 | 0.098 | 1.309 | 0.191 | 0.999 |
|  | Left Hippocampus | 0.019 | 0.092 | 0.202 | 0.840 | 0.999 |
|  | Right Hippocampus | -0.094 | 0.092 | -1.021 | 0.307 | 0.999 |
|  | WMH | -0.031 | 0.085 | -0.369 | 0.712 | 0.999 |
| **Estrogen-only Forms** |  |  |  |  |  |  |
| Bioidentical | GM BAG | 0.010 | 0.085 | 0.115 | 0.909 | 0.999 |
|  | WM BAG | 0.030 | 0.086 | 0.354 | 0.723 | 0.999 |
|  | Left Hippocampus | -0.034 | 0.081 | -0.420 | 0.674 | 0.999 |
|  | Right Hippocampus | -0.020 | 0.081 | -0.249 | 0.804 | 0.999 |
|  | WMH | 0.033 | 0.075 | 0.443 | 0.658 | 0.999 |
| Synthetic | GM BAG | 0.043 | 0.237 | 0.180 | 0.857 | 0.999 |
|  | WM BAG | 0.025 | 0.237 | 0.106 | 0.916 | 0.999 |
|  | Left Hippocampus | -0.216 | 0.224 | -0.964 | 0.335 | 0.999 |
|  | Right Hippocampus | -0.075 | 0.224 | -0.332 | 0.740 | 0.999 |
|  | WMH | 0.076 | 0.202 | 0.376 | 0.707 | 0.999 |
| **Estrogen-only,**  **active ingredient** |  |  |  |  |  |  |
| estradiol | GM BAG | -0.127 | 0.196 | -0.646 | 0.518 | 0.999 |
|  | WM BAG | 0.066 | 0.197 | 0.335 | 0.738 | 0.999 |
|  | Left Hippocampus | -0.051 | 0.186 | -0.273 | 0.785 | 0.999 |
|  | Right Hippocampus | -0.132 | 0.186 | -0.710 | 0.478 | 0.999 |
|  | WMH | 0.121 | 0.171 | 0.706 | 0.480 | 0.999 |
| estradiol hemihydrate | GM BAG | 0.041 | 0.094 | 0.431 | 0.667 | 0.999 |
|  | WM BAG | 0.022 | 0.095 | 0.238 | 0.812 | 0.999 |
|  | Left Hippocampus | -0.030 | 0.089 | -0.336 | 0.737 | 0.999 |
|  | Right Hippocampus | 0.005 | 0.089 | 0.058 | 0.954 | 0.999 |
|  | WMH | 0.013 | 0.084 | 0.158 | 0.875 | 0.999 |
| estradiol valerate | GM BAG | 0.442 | 0.498 | 0.888 | 0.375 | 0.999 |
|  | WM BAG | 0.277 | 0.500 | 0.554 | 0.579 | 0.999 |
|  | Left Hippocampus | -0.015 | 0.471 | -0.032 | 0.974 | 0.999 |
|  | Right Hippocampus | 0.378 | 0.472 | 0.801 | 0.423 | 0.999 |
|  | WMH | 0.174 | 0.425 | 0.409 | 0.682 | 0.999 |
| CEE | GM BAG | -0.074 | 0.268 | -0.277 | 0.782 | 0.999 |
|  | WM BAG | -0.046 | 0.269 | -0.173 | 0.863 | 0.999 |
|  | Left Hippocampus | -0.272 | 0.253 | -1.074 | 0.283 | 0.999 |
|  | Right Hippocampus | -0.206 | 0.254 | -0.810 | 0.418 | 0.999 |
|  | WMH | 0.050 | 0.229 | 0.217 | 0.828 | 0.999 |
| Mixed | GM BAG | -0.304 | 0.259 | -1.174 | 0.241 | 0.999 |
|  | WM BAG | 0.260 | 0.260 | 1.003 | 0.316 | 0.999 |
|  | Left Hippocampus | -0.223 | 0.245 | -0.910 | 0.363 | 0.999 |
|  | Right Hippocampus | -0.394 | 0.245 | -1.605 | 0.109 | 0.999 |
|  | WMH | 0.252 | 0.229 | 1.103 | 0.270 | 0.999 |
| **Estrogens-only,**  **Dosage (mg)** |  |  |  |  |  |  |
|  | GM BAG | 0.000 | 0.091 | 0.004 | 0.996 | 0.999 |
|  | WM BAG | -0.042 | 0.090 | -0.462 | 0.645 | 0.999 |
|  | Left Hippocampus | 0.001 | 0.084 | 0.014 | 0.989 | 0.999 |
|  | Right Hippocampus | 0.001 | 0.084 | 0.016 | 0.987 | 0.999 |
|  | WMH | 0.073 | 0.072 | 1.003 | 0.317 | 0.999 |
| **Estrogens-only,**  **Duration of Use (weeks)** |  |  |  |  |  |  |
|  | GM BAG | -0.067 | 0.100 | -0.670 | 0.504 | 0.999 |
|  | WM BAG | -0.081 | 0.104 | -0.774 | 0.441 | 0.999 |
|  | Left Hippocampus | -0.047 | 0.100 | -0.472 | 0.638 | 0.999 |
|  | Right Hippocampus | -0.024 | 0.098 | -0.242 | 0.809 | 0.999 |
|  | WMH | -0.154 | 0.078 | -1.989 | **0.049** | 0.986 |
| **Estrogens + Progestins Form** |  |  |  |  |  |  |
| Bioidentical | GM BAG | -0.253 | 0.256 | -0.985 | 0.325 | 0.999 |
|  | WM BAG | -0.004 | 0.258 | -0.017 | 0.986 | 0.999 |
|  | Left Hippocampus | 0.100 | 0.243 | 0.410 | 0.682 | 0.999 |
|  | Right Hippocampus | 0.393 | 0.244 | 1.612 | 0.107 | 0.999 |
|  | WMH | -0.044 | 0.220 | -0.199 | 0.842 | 0.999 |
| Synthetic | GM BAG | -0.166 | 0.199 | -0.834 | 0.404 | 0.999 |
|  | WM BAG | -0.014 | 0.200 | -0.069 | 0.945 | 0.999 |
|  | Left Hippocampus | 0.227 | 0.189 | 1.205 | 0.228 | 0.999 |
|  | Right Hippocampus | 0.233 | 0.189 | 1.232 | 0.218 | 0.999 |
|  | WMH | 0.020 | 0.171 | 0.115 | 0.909 | 0.999 |
| Bioidentical & Synthetic | GM BAG | -0.008 | 0.107 | -0.075 | 0.940 | 0.999 |
|  | WM BAG | 0.056 | 0.108 | 0.519 | 0.604 | 0.999 |
|  | Left Hippocampus | -0.061 | 0.101 | -0.604 | 0.546 | 0.999 |
|  | Right Hippocampus | -0.083 | 0.102 | -0.817 | 0.414 | 0.999 |
|  | WMH | 0.037 | 0.092 | 0.407 | 0.684 | 0.999 |
| **Estrogens + Progestins,**  **active ingredient** |  |  |  |  |  |  |
| estradiol hemihydrate &  norethisterone acetate | GM BAG | -0.058 | 0.142 | -0.405 | 0.685 | 0.999 |
|  | WM BAG | -0.001 | 0.143 | -0.006 | 0.995 | 0.999 |
|  | Left Hippocampus | 0.042 | 0.135 | 0.310 | 0.757 | 0.999 |
|  | Right Hippocampus | -0.013 | 0.135 | -0.094 | 0.925 | 0.999 |
|  | WMH | 0.026 | 0.122 | 0.213 | 0.832 | 0.999 |
| estradiol hemihydrate &  dydrogesterone | GM BAG | -0.362 | 0.276 | -1.315 | 0.188 | 0.999 |
|  | WM BAG | -0.121 | 0.277 | -0.438 | 0.661 | 0.999 |
|  | Left Hippocampus | 0.024 | 0.261 | 0.090 | 0.928 | 0.999 |
|  | Right Hippocampus | 0.387 | 0.261 | 1.480 | 0.139 | 0.999 |
|  | WMH | -0.105 | 0.236 | -0.447 | 0.655 | 0.999 |
| estradiol hemihydrate &  norethisterone | GM BAG | 0.287 | 0.287 | 1.001 | 0.317 | 0.999 |
|  | WM BAG | 0.193 | 0.288 | 0.669 | 0.504 | 0.999 |
|  | Left Hippocampus | -0.215 | 0.272 | -0.791 | 0.429 | 0.999 |
|  | Right Hippocampus | -0.286 | 0.272 | -1.052 | 0.293 | 0.999 |
|  | WMH | 0.369 | 0.245 | 1.506 | 0.132 | 0.999 |
| CEE & norgestrel | GM BAG | -0.116 | 0.241 | -0.480 | 0.632 | 0.999 |
|  | WM BAG | -0.210 | 0.242 | -0.866 | 0.387 | 0.999 |
|  | Left Hippocampus | 0.068 | 0.228 | 0.296 | 0.767 | 0.999 |
|  | Right Hippocampus | -0.051 | 0.229 | -0.221 | 0.825 | 0.999 |
|  | WMH | 0.098 | 0.206 | 0.474 | 0.635 | 0.999 |
| CEE &  medroxyprogesterone acetate | GM BAG | -0.267 | 0.351 | -0.761 | 0.447 | 0.999 |
|  | WM BAG | 0.403 | 0.353 | 1.141 | 0.254 | 0.999 |
|  | Left Hippocampus | 0.558 | 0.333 | 1.678 | 0.093 | 0.999 |
|  | Right Hippocampus | 0.830 | 0.333 | 2.493 | **0.013** | 0.847 |
|  | WMH | -0.147 | 0.300 | -0.490 | 0.624 | 0.999 |
| tibolone | GM BAG | -0.418 | 0.276 | -1.514 | 0.130 | 0.999 |
|  | WM BAG | 0.036 | 0.277 | 0.129 | 0.898 | 0.999 |
|  | Left Hippocampus | 0.016 | 0.261 | 0.063 | 0.950 | 0.999 |
|  | Right Hippocampus | 0.153 | 0.262 | 0.584 | 0.560 | 0.999 |
|  | WMH | -0.156 | 0.245 | -0.637 | 0.524 | 0.999 |
| Mixed | GM BAG | 0.116 | 0.083 | 1.408 | 0.159 | 0.999 |
|  | WM BAG | 0.172 | 0.083 | 2.069 | **0.039** | 0.986 |
|  | Left Hippocampus | 0.035 | 0.078 | 0.451 | 0.652 | 0.999 |
|  | Right Hippocampus | -0.030 | 0.078 | -0.383 | 0.702 | 0.999 |
|  | WMH | 0.064 | 0.072 | 0.881 | 0.378 | 0.999 |
| **Estrogens + Progestins,**  **Progestin Generation** |  |  |  |  |  |  |
| 1stGen | GM BAG | 0.035 | 0.097 | 0.364 | 0.716 | 0.999 |
|  | WM BAG | 0.136 | 0.097 | 1.393 | 0.164 | 0.999 |
|  | Left Hippocampus | 0.013 | 0.092 | 0.144 | 0.886 | 0.999 |
|  | Right Hippocampus | 0.000 | 0.092 | -0.001 | 0.999 | 0.999 |
|  | WMH | 0.061 | 0.084 | 0.722 | 0.470 | 0.999 |
| 2ndGen | GM BAG | -0.064 | 0.162 | -0.393 | 0.695 | 0.999 |
|  | WM BAG | -0.011 | 0.162 | -0.068 | 0.946 | 0.999 |
|  | Left Hippocampus | 0.044 | 0.153 | 0.288 | 0.773 | 0.999 |
|  | Right Hippocampus | 0.084 | 0.153 | 0.549 | 0.583 | 0.999 |
|  | WMH | 0.082 | 0.139 | 0.595 | 0.552 | 0.999 |
| **Estrogens + Progestins,**  **Dosage (mg)** |  |  |  |  |  |  |
| Estrogens | GM BAG | -0.075 | 0.059 | -1.264 | 0.207 | 0.999 |
|  | WM BAG | -0.003 | 0.059 | -0.044 | 0.965 | 0.999 |
|  | Left Hippocampus | -0.074 | 0.057 | -1.293 | 0.197 | 0.999 |
|  | Right Hippocampus | -0.009 | 0.057 | -0.158 | 0.875 | 0.999 |
|  | WMH | 0.047 | 0.053 | 0.887 | 0.376 | 0.999 |
| Progestins | GM BAG | 0.064 | 0.057 | 1.132 | 0.258 | 0.999 |
|  | WM BAG | 0.055 | 0.057 | 0.965 | 0.335 | 0.999 |
|  | Left Hippocampus | 0.063 | 0.055 | 1.160 | 0.247 | 0.999 |
|  | Right Hippocampus | 0.041 | 0.054 | 0.765 | 0.445 | 0.999 |
|  | WMH | 0.071 | 0.050 | 1.416 | 0.158 | 0.999 |
| **Estrogens + Progestins,**  **Duration of Use (weeks)** | |  |  |  |  |  |
| Estrogens | GM BAG | 0.161 | 0.081 | 1.984 | **0.048** | 0.986 |
|  | WM BAG | -0.018 | 0.083 | -0.214 | 0.831 | 0.999 |
|  | Left Hippocampus | -0.170 | 0.077 | -2.204 | **0.028** | 0.986 |
|  | Right Hippocampus | -0.183 | 0.075 | -2.428 | **0.016** | 0.847 |
|  | WMH | -0.105 | 0.074 | -1.432 | 0.153 | 0.999 |
| Progestins | GM BAG | -0.042 | 0.081 | -0.518 | 0.605 | 0.999 |
|  | WM BAG | 0.087 | 0.083 | 1.042 | 0.298 | 0.999 |
|  | Left Hippocampus | -0.025 | 0.077 | -0.328 | 0.743 | 0.999 |
|  | Right Hippocampus | 0.028 | 0.075 | 0.377 | 0.707 | 0.999 |
|  | WMH | 0.089 | 0.073 | 1.218 | 0.225 | 0.999 |

Significant results are highlighted in bold. False discovery rate (FDR) correction was applied across all brain measures and MHT variables listed in this table. Abbreviations: MRI = magnetic resonance imaging, S.E. = standard error, GM = grey matter, BAG = brain age gap, WM = white matter, WMH = white matter hyperintensity, CEE = conjugated equine estrogen, Gen = generation.
