# Supplementary material for: Menopausal hormone therapy and the female brain: Leveraging neuroimaging and prescription registry data from the UK Biobank cohort
Source: eLife. 2025 May 29;13:RP99538. doi: 10.7554/eLife.99538 (PMC12122002; doi:10.7554/eLife.99538)
Supplement: Supplementary file 15. [file elife-99538-supp15.docx]

**Supplemental File 15| Associations between menopausal hormone therapy (MHT)-related variables and brain measures in the prescription MHT sample, with age, education, and menopause-status matched never-users.**

| **MHT Variable** | **MRI Measure** | **beta** | **S.E.** | **t-value** | **p-value** | **pFDR-value** |
| --- | --- | --- | --- | --- | --- | --- |
| **MHT formulation** |  |  |  |  |  |  |
| Estrogens-only | GM BAG | -0.071 | 0.080 | -0.889 | 0.374 | 0.986 |
|  | WM BAG | -0.009 | 0.080 | -0.112 | 0.911 | 0.986 |
|  | Left Hippocampus | 0.008 | 0.075 | 0.106 | 0.916 | 0.986 |
|  | Right Hippocampus | -0.006 | 0.075 | -0.087 | 0.931 | 0.986 |
|  | WMH | 0.067 | 0.071 | 0.950 | 0.342 | 0.986 |
| Estrogens+Progestin | GM BAG | -0.027 | 0.071 | -0.377 | 0.706 | 0.986 |
|  | WM BAG | 0.088 | 0.071 | 1.233 | 0.218 | 0.986 |
|  | Left Hippocampus | 0.051 | 0.067 | 0.764 | 0.445 | 0.986 |
|  | Right Hippocampus | 0.019 | 0.067 | 0.280 | 0.779 | 0.986 |
|  | WMH | 0.099 | 0.063 | 1.575 | 0.115 | 0.986 |
| **Route of Administration** |  |  |  |  |  |  |
| oral | GM BAG | -0.083 | 0.078 | -1.060 | 0.289 | 0.986 |
|  | WM BAG | 0.068 | 0.079 | 0.869 | 0.385 | 0.986 |
|  | Left Hippocampus | 0.036 | 0.074 | 0.485 | 0.628 | 0.986 |
|  | Right Hippocampus | 0.034 | 0.074 | 0.459 | 0.646 | 0.986 |
|  | WMH | 0.178 | 0.069 | 2.586 | **0.010** | 0.986 |
| transdermal | GM BAG | -0.247 | 0.132 | -1.868 | 0.062 | 0.986 |
|  | WM BAG | -0.082 | 0.132 | -0.621 | 0.535 | 0.986 |
|  | Left Hippocampus | 0.032 | 0.125 | 0.254 | 0.800 | 0.986 |
|  | Right Hippocampus | -0.015 | 0.124 | -0.121 | 0.904 | 0.986 |
|  | WMH | -0.034 | 0.117 | -0.290 | 0.772 | 0.986 |
| vaginal | GM BAG | 0.101 | 0.105 | 0.958 | 0.338 | 0.986 |
|  | WM BAG | 0.104 | 0.105 | 0.987 | 0.324 | 0.986 |
|  | Left Hippocampus | 0.010 | 0.100 | 0.098 | 0.922 | 0.986 |
|  | Right Hippocampus | 0.026 | 0.099 | 0.264 | 0.792 | 0.986 |
|  | WMH | 0.118 | 0.094 | 1.251 | 0.211 | 0.986 |
| injection | GM BAG | 0.377 | 0.336 | 1.121 | 0.262 | 0.986 |
|  | WM BAG | 0.159 | 0.337 | 0.472 | 0.637 | 0.986 |
|  | Left Hippocampus | -0.191 | 0.319 | -0.598 | 0.550 | 0.986 |
|  | Right Hippocampus | 0.138 | 0.316 | 0.437 | 0.662 | 0.986 |
|  | WMH | -0.018 | 0.291 | -0.060 | 0.952 | 0.986 |
| mixed | GM BAG | -0.028 | 0.100 | -0.282 | 0.778 | 0.986 |
|  | WM BAG | 0.017 | 0.100 | 0.172 | 0.864 | 0.986 |
|  | Left Hippocampus | 0.066 | 0.095 | 0.699 | 0.485 | 0.986 |
|  | Right Hippocampus | -0.054 | 0.094 | -0.570 | 0.569 | 0.986 |
|  | WMH | -0.050 | 0.088 | -0.563 | 0.574 | 0.986 |
| **Estrogen-only Forms** |  |  |  |  |  |  |
| Bioidentical | GM BAG | -0.052 | 0.087 | -0.594 | 0.553 | 0.986 |
|  | WM BAG | 0.002 | 0.087 | 0.018 | 0.986 | 0.986 |
|  | Left Hippocampus | 0.025 | 0.082 | 0.304 | 0.761 | 0.986 |
|  | Right Hippocampus | 0.018 | 0.081 | 0.219 | 0.827 | 0.986 |
|  | WMH | 0.056 | 0.077 | 0.726 | 0.468 | 0.986 |
| Synthetic | GM BAG | -0.030 | 0.218 | -0.138 | 0.890 | 0.986 |
|  | WM BAG | -0.056 | 0.218 | -0.257 | 0.797 | 0.986 |
|  | Left Hippocampus | -0.017 | 0.205 | -0.085 | 0.932 | 0.986 |
|  | Right Hippocampus | 0.026 | 0.203 | 0.126 | 0.900 | 0.986 |
|  | WMH | 0.133 | 0.187 | 0.711 | 0.477 | 0.986 |
| **Estrogen-only, active ingredient** |  |  |  |  |  |  |
| estradiol | GM BAG | -0.284 | 0.177 | -1.606 | 0.109 | 0.986 |
|  | WM BAG | -0.186 | 0.177 | -1.051 | 0.294 | 0.986 |
|  | Left Hippocampus | 0.034 | 0.166 | 0.204 | 0.838 | 0.986 |
|  | Right Hippocampus | 0.068 | 0.164 | 0.416 | 0.677 | 0.986 |
|  | WMH | 0.003 | 0.154 | 0.018 | 0.986 | 0.986 |
| estradiol hemihydrate | GM BAG | 0.005 | 0.095 | 0.050 | 0.960 | 0.986 |
|  | WM BAG | 0.047 | 0.095 | 0.492 | 0.623 | 0.986 |
|  | Left Hippocampus | 0.022 | 0.089 | 0.251 | 0.802 | 0.986 |
|  | Right Hippocampus | 0.005 | 0.088 | 0.061 | 0.952 | 0.986 |
|  | WMH | 0.069 | 0.084 | 0.825 | 0.410 | 0.986 |
| estradiol valerate | GM BAG | 0.409 | 0.502 | 0.814 | 0.416 | 0.986 |
|  | WM BAG | 0.191 | 0.503 | 0.380 | 0.704 | 0.986 |
|  | Left Hippocampus | 0.062 | 0.471 | 0.131 | 0.896 | 0.986 |
|  | Right Hippocampus | 0.427 | 0.465 | 0.920 | 0.358 | 0.986 |
|  | WMH | 0.214 | 0.429 | 0.500 | 0.617 | 0.986 |
| CEE | GM BAG | -0.129 | 0.241 | -0.537 | 0.591 | 0.986 |
|  | WM BAG | -0.109 | 0.241 | -0.453 | 0.651 | 0.986 |
|  | Left Hippocampus | -0.032 | 0.226 | -0.144 | 0.886 | 0.986 |
|  | Right Hippocampus | -0.061 | 0.222 | -0.273 | 0.785 | 0.986 |
|  | WMH | 0.115 | 0.206 | 0.558 | 0.577 | 0.986 |
| Mixed | GM BAG | -0.255 | 0.198 | -1.292 | 0.197 | 0.986 |
|  | WM BAG | -0.047 | 0.198 | -0.236 | 0.813 | 0.986 |
|  | Left Hippocampus | -0.066 | 0.185 | -0.354 | 0.724 | 0.986 |
|  | Right Hippocampus | -0.168 | 0.183 | -0.917 | 0.360 | 0.986 |
|  | WMH | 0.075 | 0.172 | 0.437 | 0.662 | 0.986 |
| **Estrogens + Progestins Form** |  |  |  |  |  |  |
| Bioidentical | GM BAG | -0.148 | 0.247 | -0.598 | 0.550 | 0.986 |
|  | WM BAG | -0.023 | 0.261 | -0.087 | 0.931 | 0.986 |
|  | Left Hippocampus | 0.143 | 0.241 | 0.594 | 0.553 | 0.986 |
|  | Right Hippocampus | 0.317 | 0.243 | 1.303 | 0.193 | 0.986 |
|  | WMH | 0.080 | 0.224 | 0.356 | 0.722 | 0.986 |
| Synthetic | GM BAG | -0.050 | 0.187 | -0.270 | 0.787 | 0.986 |
|  | WM BAG | 0.149 | 0.197 | 0.753 | 0.452 | 0.986 |
|  | Left Hippocampus | 0.220 | 0.182 | 1.208 | 0.228 | 0.986 |
|  | Right Hippocampus | 0.235 | 0.184 | 1.277 | 0.202 | 0.986 |
|  | WMH | 0.177 | 0.170 | 1.046 | 0.296 | 0.986 |
| Bioidentical & Synthetic | GM BAG | -0.027 | 0.111 | -0.247 | 0.805 | 0.986 |
|  | WM BAG | 0.023 | 0.117 | 0.192 | 0.848 | 0.986 |
|  | Left Hippocampus | -0.052 | 0.108 | -0.477 | 0.634 | 0.986 |
|  | Right Hippocampus | -0.112 | 0.110 | -1.019 | 0.309 | 0.986 |
|  | WMH | 0.120 | 0.102 | 1.177 | 0.240 | 0.986 |
| **Estrogens + Progestins,**  **active ingredient** |  |  |  |  |  |  |
| estradiol hemihydrate &  norethisterone acetate | GM BAG | -0.099 | 0.146 | -0.679 | 0.497 | 0.986 |
|  | WM BAG | -0.044 | 0.148 | -0.299 | 0.765 | 0.986 |
|  | Left Hippocampus | 0.095 | 0.140 | 0.677 | 0.499 | 0.986 |
|  | Right Hippocampus | 0.003 | 0.139 | 0.018 | 0.986 | 0.986 |
|  | WMH | 0.112 | 0.129 | 0.870 | 0.385 | 0.986 |
| estradiol hemihydrate &  dydrogesterone | GM BAG | -0.224 | 0.269 | -0.833 | 0.405 | 0.986 |
|  | WM BAG | -0.120 | 0.272 | -0.439 | 0.660 | 0.986 |
|  | Left Hippocampus | 0.086 | 0.257 | 0.336 | 0.737 | 0.986 |
|  | Right Hippocampus | 0.320 | 0.255 | 1.251 | 0.211 | 0.986 |
|  | WMH | 0.042 | 0.235 | 0.178 | 0.859 | 0.986 |
| estradiol hemihydrate &  norethisterone | GM BAG | 0.222 | 0.280 | 0.794 | 0.428 | 0.986 |
|  | WM BAG | 0.099 | 0.283 | 0.351 | 0.726 | 0.986 |
|  | Left Hippocampus | -0.308 | 0.267 | -1.152 | 0.250 | 0.986 |
|  | Right Hippocampus | -0.426 | 0.265 | -1.605 | 0.109 | 0.986 |
|  | WMH | 0.407 | 0.244 | 1.667 | 0.096 | 0.986 |
| CEE & norgestrel | GM BAG | -0.098 | 0.234 | -0.418 | 0.676 | 0.986 |
|  | WM BAG | -0.102 | 0.236 | -0.434 | 0.664 | 0.986 |
|  | Left Hippocampus | 0.212 | 0.223 | 0.951 | 0.342 | 0.986 |
|  | Right Hippocampus | 0.110 | 0.222 | 0.496 | 0.620 | 0.986 |
|  | WMH | 0.166 | 0.204 | 0.816 | 0.415 | 0.986 |
| CEE &  medroxyprogesterone acetate | GM BAG | 0.042 | 0.318 | 0.134 | 0.894 | 0.986 |
|  | WM BAG | 0.566 | 0.321 | 1.765 | 0.078 | 0.986 |
|  | Left Hippocampus | 0.198 | 0.303 | 0.652 | 0.515 | 0.986 |
|  | Right Hippocampus | 0.440 | 0.301 | 1.461 | 0.145 | 0.986 |
|  | WMH | 0.167 | 0.277 | 0.601 | 0.548 | 0.986 |
| tibolone | GM BAG | -0.446 | 0.282 | -1.582 | 0.114 | 0.986 |
|  | WM BAG | -0.069 | 0.285 | -0.241 | 0.810 | 0.986 |
|  | Left Hippocampus | 0.135 | 0.269 | 0.500 | 0.617 | 0.986 |
|  | Right Hippocampus | 0.264 | 0.267 | 0.988 | 0.323 | 0.986 |
|  | WMH | -0.154 | 0.255 | -0.602 | 0.547 | 0.986 |
| Mixed | GM BAG | 0.032 | 0.089 | 0.355 | 0.722 | 0.986 |
|  | WM BAG | 0.136 | 0.090 | 1.513 | 0.131 | 0.986 |
|  | Left Hippocampus | 0.058 | 0.085 | 0.681 | 0.496 | 0.986 |
|  | Right Hippocampus | -0.004 | 0.084 | -0.042 | 0.966 | 0.986 |
|  | WMH | 0.084 | 0.079 | 1.063 | 0.288 | 0.986 |
| **Estrogens + Progestins,**  **Progestin Generation** |  |  |  |  |  |  |
| 1stGen | GM BAG | 0.019 | 0.104 | 0.179 | 0.858 | 0.986 |
|  | WM BAG | 0.101 | 0.104 | 0.977 | 0.329 | 0.986 |
|  | Left Hippocampus | 0.007 | 0.097 | 0.067 | 0.946 | 0.986 |
|  | Right Hippocampus | -0.029 | 0.096 | -0.299 | 0.765 | 0.986 |
|  | WMH | 0.130 | 0.093 | 1.391 | 0.165 | 0.986 |
| 2ndGen | GM BAG | -0.035 | 0.162 | -0.216 | 0.829 | 0.986 |
|  | WM BAG | 0.021 | 0.162 | 0.128 | 0.898 | 0.986 |
|  | Left Hippocampus | 0.148 | 0.152 | 0.973 | 0.331 | 0.986 |
|  | Right Hippocampus | 0.142 | 0.151 | 0.939 | 0.348 | 0.986 |
|  | WMH | 0.164 | 0.144 | 1.138 | 0.255 | 0.986 |

Significant results are highlighted in bold. False discovery rate (FDR) correction was applied across all brain measures and MHT variables listed in this table. Abbreviations: MRI = magnetic resonance imaging, S.E. = standard error, GM = grey matter, BAG = brain age gap, WM = white matter, WMH = white matter hyperintensity, CEE = conjugated equine estrogen, Gen = generation.
